# Grape berry ripening delay induced by a pre-véraison NAA treatment is paralleled by a shift in the expression pattern of auxin- and ethylene-related genes

**DOI:** 10.1186/1471-2229-12-185

**Published:** 2012-10-09

**Authors:** Fiorenza Ziliotto, Massimiliano Corso, Fabio Massimo Rizzini, Angela Rasori, Alessandro Botton, Claudio Bonghi

**Affiliations:** 1 Department of Agronomy, Food, Natural resources, Animals and Environment, DAFNAE, University of Padova, Agripolis – Viale dell’Università 16, 35020, Legnaro, Padova, Italy; 2 Centro Interdipartimentale per la Ricerca in Viticoltura ed Enologia, CIRVE, University of Padova, Agripolis – Viale dell’Università 16, 35020, Legnaro, Padova, Italy

**Keywords:** *Vitis vinifera*, Hormones, Cross-talk, Fruit development, Microarray, HORMONOMETER, Transcriptomics

## Abstract

**Background:**

Auxins act as repressors of ripening inception in grape (véraison), while ethylene and abscisic acid (ABA) play a positive role as inducers of the syndrome. Despite the increasing amount of information made available on this topic, the complex network of interactions among these hormones remains elusive. In order to shed light on these aspects, a holistic approach was adopted to evaluate, at the transcriptomic level, the crosstalk between hormones in grape berries, whose ripening progression was delayed by applying naphtalenacetic acid (NAA) one week before véraison.

**Results:**

The NAA treatment caused significant changes in the transcription rate of about 1,500 genes, indicating that auxin delayed grape berry ripening also at the transcriptional level, along with the recovery of a steady state of its intracellular concentration. Hormone indices analysis carried out with the HORMONOMETER tool suggests that biologically active concentrations of auxins were achieved throughout a homeostatic recovery. This occurred within 7 days after the treatment, during which the physiological response was mainly unspecific and due to a likely pharmacological effect of NAA. This hypothesis is strongly supported by the up-regulation of genes involved in auxin conjugation (*GH3-like*) and action (*IAA4-* and *IAA31-like*). A strong antagonistic effect between auxin and ethylene was also observed, along with a substantial ‘synergism’ between auxins and ABA, although to a lesser extent.

**Conclusions:**

This study suggests that, in presence of altered levels of auxins, the crosstalk between hormones involves diverse mechanisms, acting at both the hormone response and biosynthesis levels, creating a complex response network.

## Background

A large number of physiological and molecular events are known to occur during grape berry ripening, but the regulatory mechanisms controlling this critical developmental phase are still poorly understood. The onset of ripening (termed véraison) is accompanied by significant changes, at both physical (pulp firmness) and chemical (accumulation of sugars and flavor compounds, synthesis of anthocyanins and reduction of organic acids concentration) levels [1,2], concurrently with the modification of the transcription rate of a large number of related genes [3,4].

Auxin, ethylene, abscisic acid (ABA) and brassinosteroids (BRs) are actively involved, throughout a complex network of interactions with other mobile signals, in the regulation of grape berry ripening [5]. Interestingly, the highest levels of auxin are observed at early berry development, then its concentration decreases rapidly before véraison, becoming undetectable after two weeks [6,7]. On the other hand, another study showed no dramatic changes in auxin concentration during berry growth and development [8]. Application of synthetic auxins before véraison delays ripening, as seen in several ripening-related physiological processes [7,9,10], and heavily modifies the transcription of key genes involved in the sugars metabolism, cell wall turn-over and biosynthesis of phenylpropanoids [11]. Among the latter, the expression of genes encoding chalcone synthase (*CHS*), flavanone 3-hydroxylase (*F3H*), UDP-glucose:flavonoid 3-O-glucosyltransferase (*UFGT*), and MYB transcription factors [5,9] is negatively affected by auxin. Davies et al. [9] showed that treatments with the synthetic auxin BTOA (benzothiazole-2-oxyacetic acid) were able to modify the hexose accumulation mechanisms by altering the expression of the related genes. NAA applications at véraison also inhibited genes belonging to cell wall structure, such as *GRIP4* coding for a proline-rich protein, and negatively affected ABA metabolism [5].

Endogenous levels of ethylene, ABA and BRs increase at véraison, and exogenous applications of these hormones accelerate the initiation of the ripening phase, concurrently stimulating the accumulation of anthocyanins, most likely by enhancing the transcription of *CHS, F3H, UFGT,* and *MYB1* genes [8,11-14]. These treatments can also induce the uptake and storage of sugars by berries [13]. In addition, low doses of ethylene at véraison stimulated grape berry expansion, enabling cell elongation in pulp and skin, and inducing genes encoding aquaporins (*AQUAPORIN1* and *AQUAPORIN2*) and cell wall hydrolase/esterase, such as Polygalacturonase (*PG1*), Expansin (*EX*), and Pectin-methyl esterase (*PME*) [12,15].

Since mutants with impaired ripening are not available in grapevine, the best alternative way to investigate the role of hormones during berry development consists in altering the specific process by means of exogenous applications of plant growth regulators. Transcriptome studies dealing with the effects of exogenous hormone treatments in grapevine have focused on ethylene [12], referring to the pivotal role of this hormone in the transcriptional regulation of its biosynthesis and signal transduction during grape berry development. In particular, ethylene treatments were shown to induce the transcription of *ARF8* (*auxin response factor*) and *NCED* (*9-cis-epoxycarotenoid dioxygenase*) genes, the latter encoding a key enzyme of ABA biosynthesis [12]. Auxin treatments were also investigated, showing an increase in ethylene due to the stimulation of the expression of genes encoding its biosynthetic key enzymes [16,17] and signal transduction elements [18].

In order to shed light on the hormone interactions occurring at véraison, a specific transcriptomic study was carried out on NAA treated berries. This study confirms the capacity of NAA to delay grape berry ripening at the transcriptional level. The duration of this delay may be associated with the recovery of a steady state of auxin concentration. In the presence of altered levels of auxin, the crosstalk between hormones involves diverse specific mechanisms, acting at both the hormone response and biosynthesis levels, thus creating a complex network of transcriptional responses.

## Results

### Biochemical analyses

**Figure 1:**
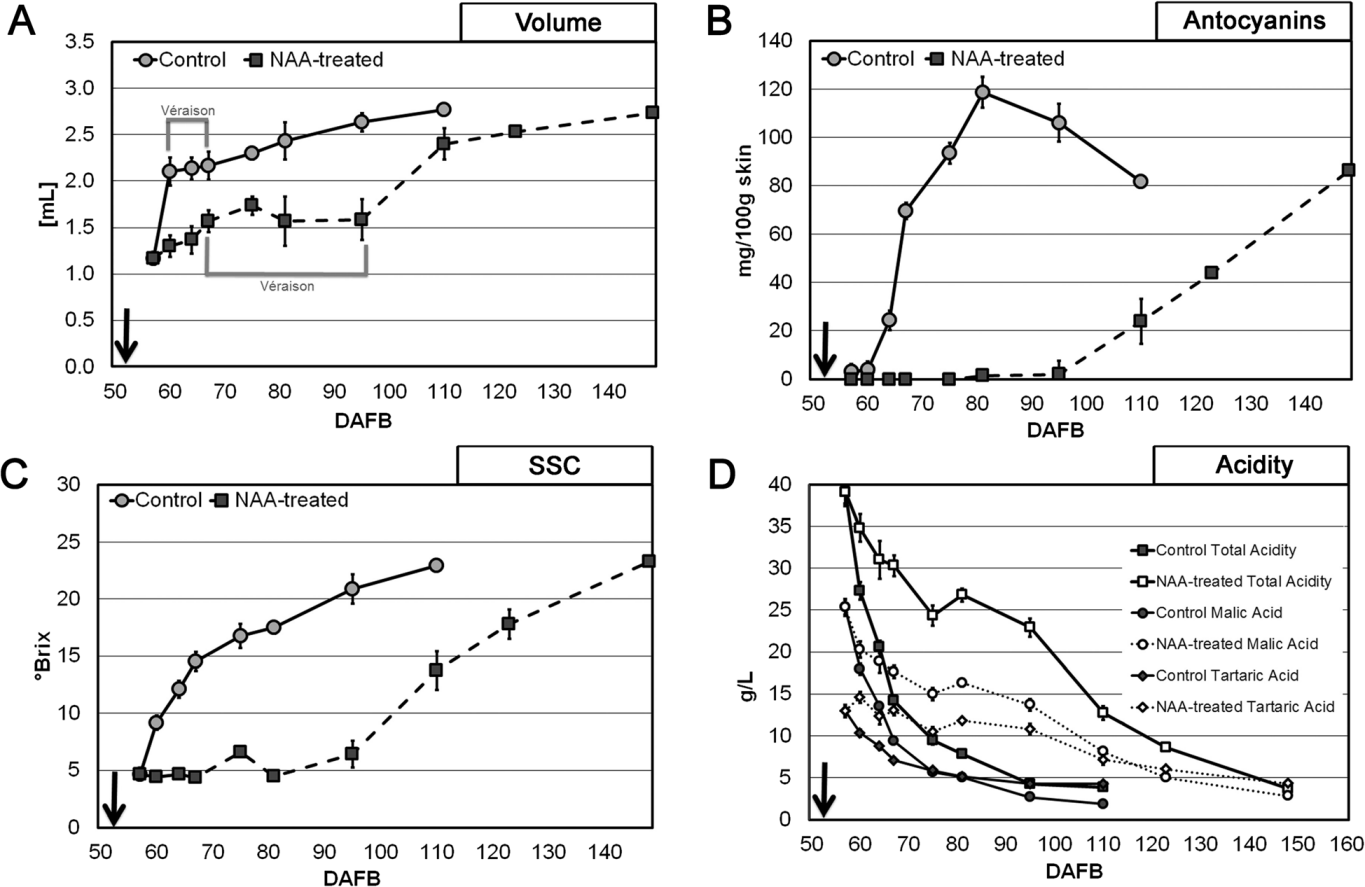
**Biochemical analysis.** Evolution of physical (berry volume) and chemical (anthocyanins content, solid soluble concentration, titratable acidity) parameters in control (circle) and NAA-treated (square) berries throughout fruit development. NAA treatment (arrow) was performed at 53 DAFB. Data concerning volume are the average of values obtained by fifty berries. Soluble solid concentration, tritatable acidity, malic acid, tartaric acid and anthocyanin contents are given by the average values of three biological replicates. Bars represent the SE.

Physical (berry volume) and chemical (total anthocyanins content, soluble solids concentration, titratable acidity) parameters were assessed in both control and NAA-treated berries (Figure 1), in order to verify the actual efficacy of the treatment.

Untreated berries showed an increase of volume after the time of the treatment (53 Days After Full Bloom, DAFB) until reaching a temporary lag phase (from 60 to 70 DAFB) during which this parameter did not vary significantly. Thereafter, it increased and reached its maximum at harvest (110 DAFB). The volume of NAA-treated berries showed a significant increase up to 70 DAFB, when their lag phase began. At this time, the volume of treated samples was about half that of the control. Moreover, the lag phase of NAA-treated berries was more than 50% longer (from 70 to 95 DAFB) with respect to the control. Thereafter, the volume increased, until reaching, at harvest (148 DAFB), a value similar to that observed in untreated samples (Figure 1A).

Anthocyanins content of whole berries in control samples increased very rapidly up to five days following the lag phase, and then gradually decreased until harvest (110 DAFB). The accumulation of anthocyanins was significantly inhibited in NAA-treated berries to almost undetectable levels up to 80 DAFB. Thereafter, a constant increase was observed until harvest (148 DAFB), finally reaching a level similar to that measured in the control (Figure 1B).

Soluble solids concentration (SSC) of control berries constantly increased throughout the whole experiment, especially during the lag phase of berry growth. NAA treatment showed an inhibitory effect also on this parameter, similar to that of anthocyanins. In fact, no increase of SSC was observed up to 80 DAFB, whereas a constant rise was measured thereafter, reaching at harvest the same values as the control (Figure 1C).

In control berries, a constant decrease of titratable acidity, well correlated with both malic and tartaric acid degradation, was observed during ripening evolution. On the other hand, NAA-treated berries always showed significantly higher levels of total acidity than the control, except for samples at harvest, whose acidity was similar to that assessed in untreated samples. However, in treated berries a clear correlation was observed only between titratable acidity and malic acid content. Additionally, a significant increase of tartaric acid was observed immediately after the NAA treatment (65 DAFB), followed by a constant but less rapid decrease compared to control fruit (Figure 1D).

### Differentially expressed genes and enrichment analysis

Three comparisons were carried out by means of microarray experiments. The samples to be compared were chosen in order to achieve as much information as possible about the effect of the auxin treatment at the transcriptional level, its duration, and the implications in terms of physiological changes and technological relevance (see Additional file 1A and B). The first comparison was carried out between NAA-treated and control fruits at 60 DAFB (N1/C1) in order to identify genes differentially expressed at 3 days after the auxin treatment, in correspondence with the onset of véraison in the control. The second comparison was made on NAA-treated and control berries at 110 DAFB (N2/C2) in correspondence of the harvest of untreated berries, to point out the effects of the treatment on ripening evolution. The third comparison (N3/C2) highlighted transcriptional differences present in treated berries, which had biochemical and phenotypic parameters similar to the control at harvest.

Among the three comparisons, genes with significant (*P* < 0.05) differential expression were 1,511 in N1/C1, 1,016 in N2/C2, and 1,136 in N3/C2 (see Additional file 2). Among the genes differentially expressed in N1/C1, N2/C2, and N3/C2, 239 (15.8%), 289 (28.4%), and 74 (6.5%) genes, respectively, showed a fold-change variation of at least 2-fold in terms of down- or up-regulation. It is noteworthy that treated samples at harvest (148 DAFB) showed an almost complete transcriptional recovery with respect to the control at 110 DAFB.

Microarray data were validated by means of qPCR experiments performed on a subset of selected genes, revealing similar expression patterns as confirmed by the significant correlation (Pearson coefficient = 0.77; *P* = 0.0007) pointed out between them (see Additional file 3).

In order to functionally classify the genes affected by the auxin treatment, Gene Ontology (GO) term enrichment analysis was performed, as described by Blüthgen et al. [19] and Botton et al. [20], in each of the three comparisons against the whole array background. A complete list of the enriched GO terms resulted from Fisher’s exact test can be found in Additional file 4, Additional file 5 and Additional file 6. In the first comparison (N1/C1), no significant enrichment was found when a *Q* < 0.05 was considered as a threshold value, although GO terms related to protein synthesis (ribonucleoprotein complex, translation, ribosome, ribonucleoprotein complex biogenesis, ribosome biogenesis, structural constituent of ribosome) were significantly over-represented (*P* < 0.01). It is noteworthy that also the terms “protein transport” and “establishment of protein localization” were those with a higher significance and shown to be under-represented. At the second comparison (N2/C2), few terms showed a significant *Q*. However, considering the *P* < 0.01, terms related to the cell wall (external encapsulating structure organization, cellular cell wall organization or biogenesis) appeared to be over-represented (Additional file 5). In the last comparison (N3/C2), GO terms related to development (developmental process, anatomical structure development, multicellular organismal development) were significantly over-represented with *Q* < 0.05. It is worthy to note that among the terms with a significant *P* value, particularly enriched are those related to 1,3-β–glucan (1,3-beta-glucan biosynthetic process, beta-glucan metabolic process, beta-glucan biosynthetic process, 1,3-beta-glucan metabolic process, 1,3-beta-glucan synthase activity, 1,3-beta-glucan synthase complex). Among the hormone-related terms, the “jasmonic acid mediated signaling pathway” was over-represented.

### MapMan analysis

To investigate the main metabolic pathways affected by the NAA treatment, a MapMan analysis [21] was performed on N1/C1 comparison based upon differentially expressed genes chosen according to *P* < 0.084, which was shown to be an acceptable threshold according to array validation analyses carried out with qPCR. This specific threshold was chosen in order to enlarge the number of genes to be used as input data for the MapMan software.

**Figure 2:**
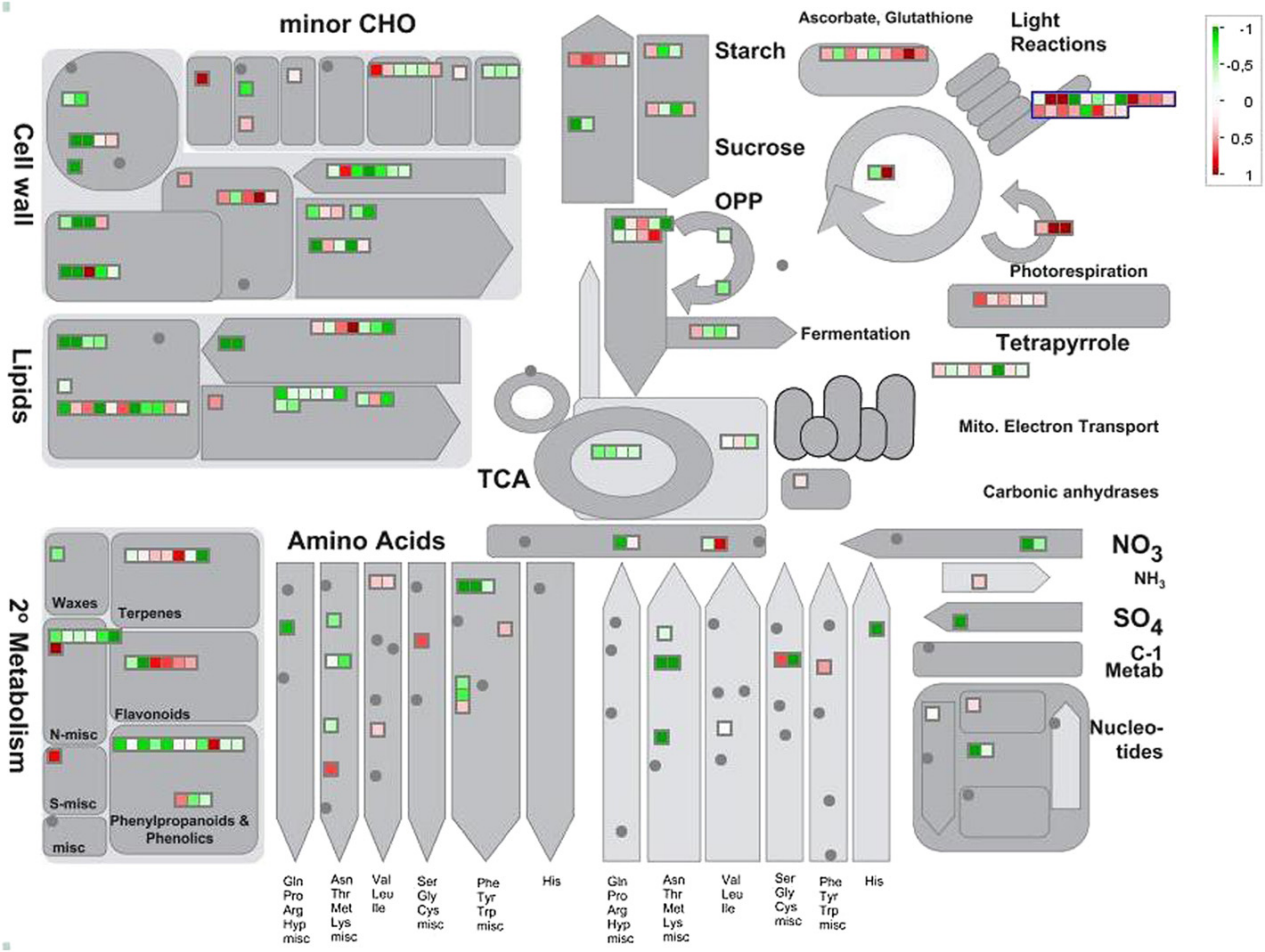
**MapMan analysis.** MapMan visualization of differences in expression of genes involved in metabolic processes. Classification into bin categories were done by using a mapping file of the grape AROS V1.0 platform (http://mapman.gabipd.org/web). Heat maps show genes with statistically significant (*P* value < 0.084) differential expression identified by comparing NAA-treated and control berries at 60 DAFB (N1/C1). A conventional red-to-green scale was used to indicate up-regulation (red) or down-regulation (green).

MapMan pointed out that several metabolisms were down-regulated in NAA-treated berries, such as those involving cell wall metabolism, carbohydrates, lipids, secondary metabolites, and amino acids, with the only exception of the light reactions pathway that showed a general up-regulation (Figure 2).

The cell wall and secondary metabolism bin categories, which were linked to the above described biochemical parameters, were investigated. The cell wall category included genes coding for pectin methyl esterase, endo-transglycosylase, polygalacturonase, and expansin-like protein (Figure 2), whereas the secondary metabolism included genes encoding alcohol dehydrogenase, phenylalanine ammonium lyase (phenylpropanoids and phenolics pathway) and chalcone synthase (flavonoid pathway). Within this secondary metabolism category, genes coding for ß-carotene hydroxylase (terpenes pathway) and cynnamoyl-CoA reductase (flavonoid pathway) showed an up-regulation in NAA-treated berries (Figure 2). Expression patterns of key genes involved in cell expansion and phenylpropanoids pathway were validated in qPCR experiments carried out in all samples (see Additional file 7). This validation analysis pointed out that the expression profiles of selected genes (anthocyanins: *CHS1*, Vv_10010748; *CHS3*, Vv_10004167; *F3H*, Vv_10003855; *UFGT*, Vv_10004481, *MYB31*, Vv17s0000g06190 and *MYB4*, Vv4s0023g03710; cell wall metabolism: PG1, Vv_10003791, and EX1, Vv_10000426; water uptake: *TIP;2-like*, Vv_10003817 and *AQUA1*, Vv_10003711), paralleled the kinetics of anthocyanins content and berry volume (Figure 1), showing an early inhibitory effect of the auxin treatment, followed by a recovery at harvest, when the treated samples showed transcripts levels similar to the control.

A detailed list of genes with the respective bin codes belonging to each MapMan category is reported in Additional file 8.

### HORMONOMETER analysis

**Figure 3:**
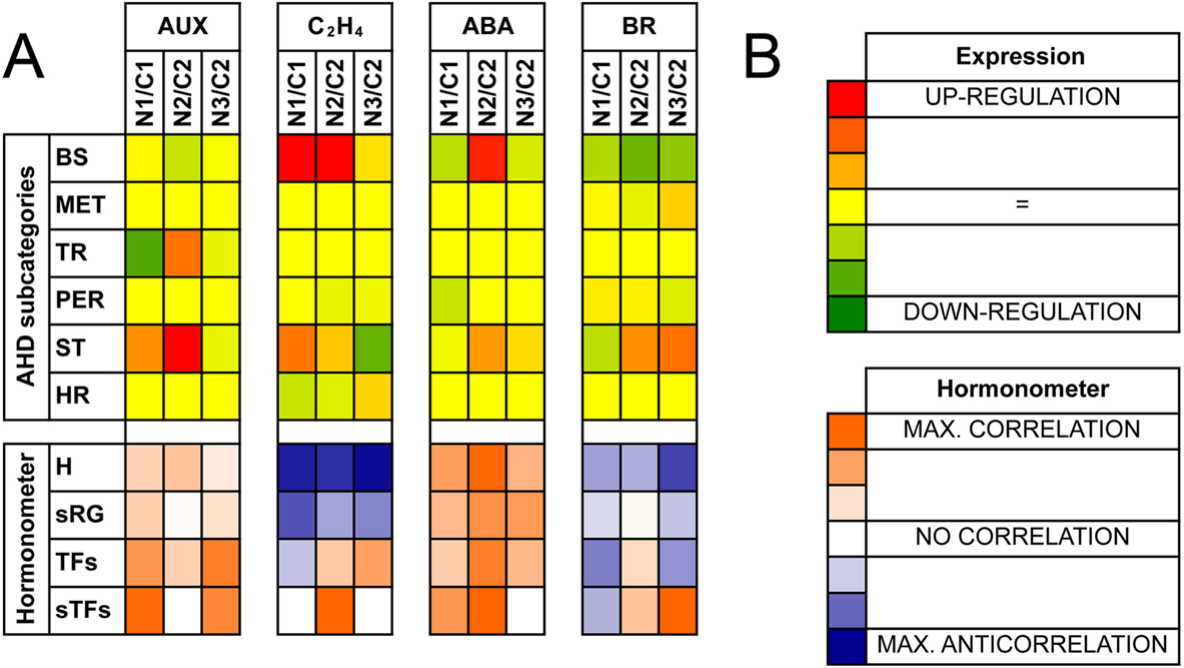
**HORMONOMETER.** Heat maps showing the expression of AHD subcategories (top) and the HORMONOMETER results (down). **A**. The heat map was produced by considering the genes encoding elements of hormone biosynthesis (BS), metabolism (MET), transport (TR), perception (PER), signal transduction (ST), and response (HR), for auxin (AUX), ethylene (C2H4), abscisic acid (ABA) and brassinosteroids (BR), which are the hormone primarily involved in grape berry ripening. HORMONOMETER data were grouped into hormone-responsive genes (H), genes with hormone-specific responsiveness (sRG), hormone-responsive genes encoding TFs (TFs), and genes encoding TFs with hormone-specific responsiveness (sTFs). For each hormone, the following comparisons have been analyzed: N1/C1, N2/C2, and N3/C2. See the Materials and Methods section for a detailed description. **B**. Colour codes for the two heat maps. For the AHD subcategories, red and green represent up- and down-regulation, respectively. In the HORMONOMETER, orange (value = 1), white (value = 0), and blue (value = -1) indicate a complete correlation, no correlation, or anti-correlation, respectively, in terms of direction and intensity of the hormone index with the queried experiment.

To understand the hormone-related transcriptional response of the berry to the auxin treatment, a HORMONOMETER analysis was carried out relying upon putative hormone indexes whose transcript levels were measured by means of the microarray. This tool allows to describe, in terms of correlation (or anti-correlation), the similarity (or dissimilarity) between a query transcriptional response and a transcriptional response typically assessed upon a certain hormone treatment as defined by known hormone indexes in Arabidopsis. Separate runs of this tool were carried out with different subsets of genes as input, as performed by Bonghi et al. [22]. The subsets are: i) all the hormone indexes (H), ii) genes with hormone-specific responsiveness (sRG), iii) hormone-responsive genes encoding TFs (TFs), and iv) genes encoding TFs with hormone-specific responsiveness (sTFs). Along with this analysis, mean log ratios (weighted according to the *P* level of significance) of genes belonging to biosynthesis (BS), metabolism (MET), transport (TR), perception (PER), signal transduction (ST) and hormone-responsiveness (HR) categories were calculated for each of the eight hormones considered by the HORMONOMETER. The categorization was made according to the Arabidopsis Hormone Database 2.0 (AHD) web site (http://ahd.cbi.pku.edu.cn/). Both analyses were carried out in the three comparisons made with microarrays and the resulting heat maps were focused on hormones involved in grape berry ripening with a primary role (i.e. auxin, ethylene, abscisic acid and brassinosteroids) (Figure 3).

The proportion of hormone responsive genes in Arabidopsis ranges between 3.8 and 9.4% of the whole transcriptome (TAIR 10 version; 27,416 genes) according to the hormone considered, whereas in grape the percentage ranges between 5.5 and 10.1% of the whole gene set (12X genome assembly, see Materials and Methods section). As far as the grape microarray is concerned (14,562 genes), the proportion of hormone responsive genes are similar to that of Arabidopsis, ranging from 4.3 to 8.9% with values for each hormone comparable to those calculated for Arabidopsis (See Additional file 9). A minimal bias may therefore be assumed to exist when grape expression data are used as input for HORMONOMETER, as hypothesized in a recent work on peach [22].

Within the AHD subcategories related to auxin, significant variations in genes encoding TR and ST elements were observed. In the first comparison (N1/C1), the auxin treatment repressed the transport of the hormone, at least at the transcriptional level, along with the significant up-regulation of its ST elements. The other AHD subcategories did not show any significant variations. These data were paralleled by a substantial correlation in the HORMONOMETER results, more significant when only the TFs were considered in the analysis, especially the auxin-specific ones (sTFs). The second comparison (N2/C2) reflected a situation typical of an auxin-related transcriptional response. The AHD subcategories indicated that the BS elements were slightly repressed and that both the TR- and ST-related genes were significantly up-regulated. This may be interpreted as a typical homeostatic response, confirmed by the HORMONOMETER results, which indicated a general unspecific correlation between auxin target expression and the typical auxin-related transcriptional response. In the last comparison (N3/C2), the AHD categories were very stable and the HORMONOMETER analysis still pointed out an active transcriptional response to auxin, with a significant correlation for the TFs and sTFs subsets, suggesting that the response to the hormone may have involved mainly auxin-specific transcription factors.

As concerns ethylene, interesting data were observed regarding both the AHD subcategories and the HORMONOMETER results. As far as the biosynthetic genes are concerned, a strong up-regulation was found in the second comparison (N2/C2), whereas data in the other two cases were less significant. The ST-related transcription showed a significant variation in all comparisons, being stimulated in the first and second (N1/C1 and N2/C2, respectively), and repressed in the third (N3/C2). Significant variations were observed also in the HR genes, which were down-regulated in all cases except the N3/C2 comparison. The HORMONOMETER analysis showed a strong and broad anti-correlation in all situations when all genes and the ethylene-specific ones (sRG) were considered. An almost reversed situation was observed in the other subsets (TFs and sTFs), except for the first comparison (N1/C1) that still showed an anti-correlation and no correlation, for TFs and sTFs, respectively. In N2/C2, a stronger correlation was found for sTFs than for TFs, whereas in the third comparison no correlation was found for the hormone-specific TFs.

Genes coding for BS elements of abscisic acid (ABA), were down-regulated at N1/C1 comparison, while in the N2/C2 comparison they were up-regulated. A weak transcriptional repression was found for genes encoding PER elements in the first comparisons, although with low significance. A stimulation of transcription was found in ST-related genes that paralleled that of BS. The HORMONOMETER showed a general correlation in all subsets and all situations, without, however, any ABA-specificity.

The brassinosteroids category showed significant data in both the analyses (AHD subcategories and HORMONOMETER). BS-related genes varied significantly in all three comparisons, with a down-regulation trend in all cases. Slight, but not significant, variations were also observed with respect to the genes encoding MET elements. A down-regulation was reported for genes coding for PER elements in the third comparison (N3/C2). Genes related to ST were down-regulated in N1/C1 and clearly up-regulated in all the other cases. Finally, the HORMONOMETER analysis evidenced an extensive anti-correlation, with the only exceptions of all TFs and sTFs in the second and third comparisons, respectively. In particular, the latter case pointed out a significant correlation.

### Expression of auxin-, ethylene-, and abscisic acid-related genes

**Figure 4:**
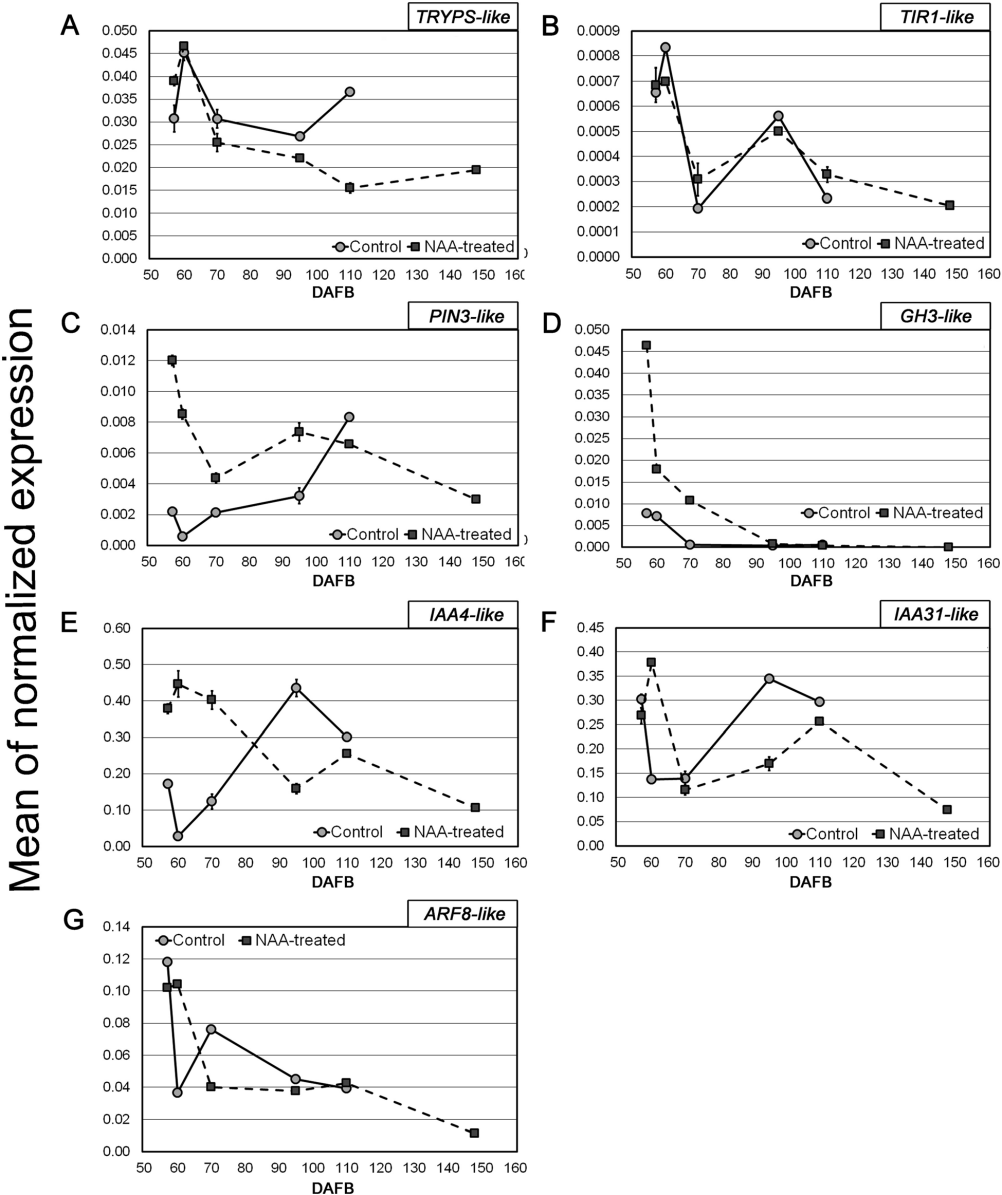
**Expression pattern, evaluated by qPCR, of genes involved in auxin biosynthesis, conjugation, transport and signal transduction.** Expression pattern, evaluated by qPCR, of the following auxin-related genes: *TRYPS-like* (Vv_1007514, **A**), *TIR1-like* (Vv_10005087, **B**), *PIN3-like* (Vv_10007217, **C**), *GH3-like* (Vv_10007966, **D**), *IAA4-like* (Vv_10002615, **E**), *IAA31-like* (Vv_10000794, **F**), *ARF8-like* (Vv_10003009, **G**). Transcript levels in NAA-treated (square) and control (circle) berries are shown as means of normalized expression ±SE.

Expression patterns of selected auxin-, ethylene-, and ABA-related genes were validated by qPCR experiments. As far as the former genes are concerned (Figure 4), the NAA treatment negatively affected the expression of *Tryptophan Synthase beta-subunit 1* (*TRYPS-like*, Figure 4A), a gene involved in the biosynthesis of tryptophan, an auxin precursor. The treatment also induced the accumulation, up to 95 DAFB, of transcripts of genes responsible for auxin perception (*Transport inhibitor response 1, TIR1-like*; Figure 4B), polar transport (*PIN3-like*; Figure 4C) and irreversible conjugation (*Indole-3-acetic acid amido synthetase*, *GH3-like*; Figure 4D). Concerning the signal transduction, two *AUX/IAA* genes (*IAA4-like* and *IAA31-like*; Figure 4E and F) and *Auxin response factor 8* (*ARF8-like*; Figure 4G) were up-regulated in treated berries one week after NAA application (60 DAFB), whereas later on and up to harvest the accumulation of their transcripts was higher in control berries.

**Figure 5:**
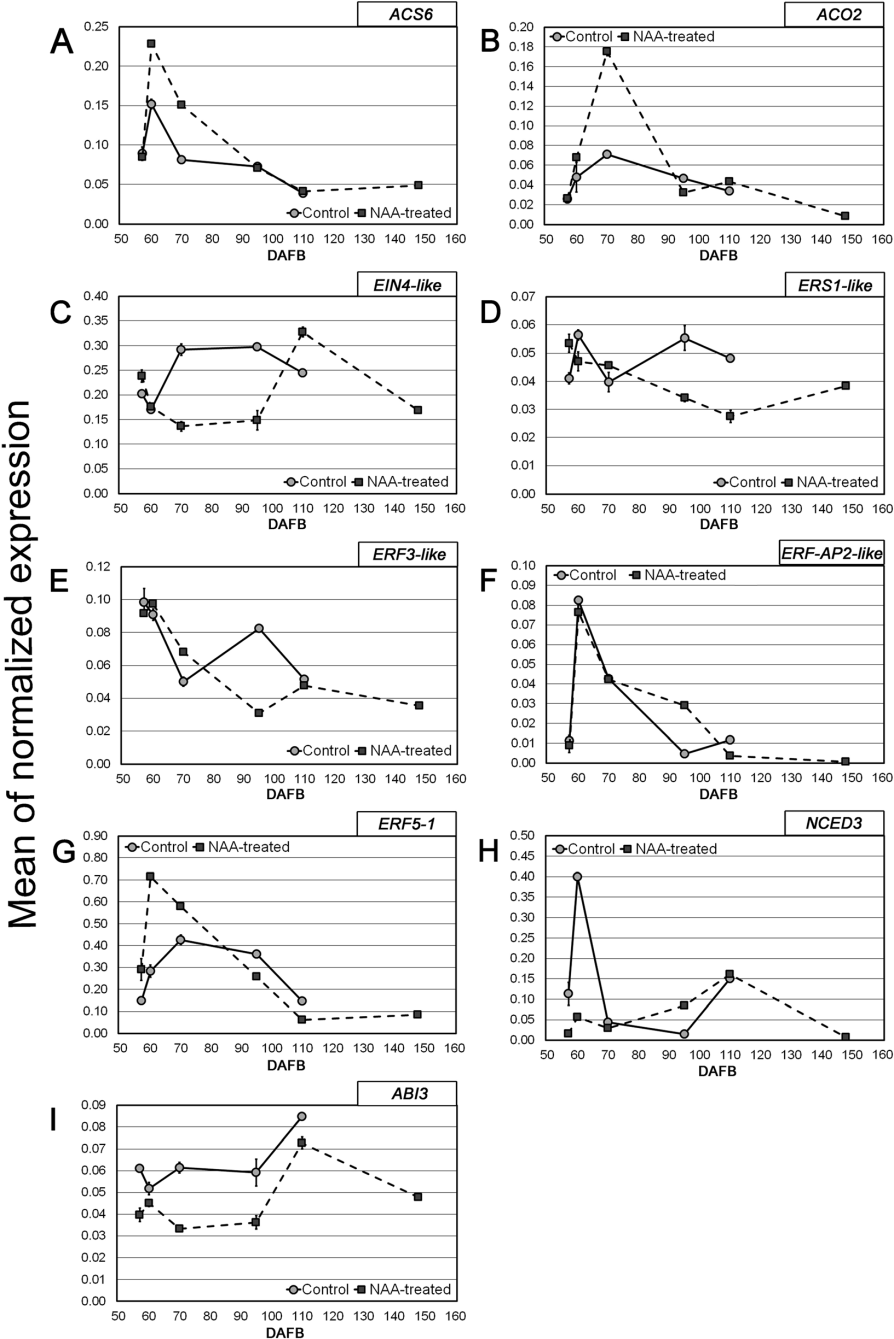
**Expression pattern, evaluated by qPCR, of genes involved in ethylene and ABA biosynthesis and signal transduction.** Expression pattern, evaluated by qPCR, of the following ethylene- and ABA-related genes: *ACS6* (Vv_10001614), *ACO2* (Vv_10004370), *EIN4-like* (Vv_10010357), *ERS1-like* (Vv_10007917), *ERF3-like* (Vv_10001775), *ERF-AP2-like* (Vv_10000332), *ERF5-1* (Vv_10001287), *NCED3* (Vv_10009127), *ABI3* (Vv_10001065). Transcript levels in NAA-treated (square) and control (circle) berries are shown as means of normalized expression ±SE.

*ACC synthase* (*ACS6*) and *ACC oxidase* (*ACO2*) genes, encoding the key enzymes of ethylene biosynthesis, were strongly up-regulated in treated berries during véraison (Figure 5A and B). Two genes encoding ethylene receptors, i.e. *Ethylene insensitive 4* (*EIN4-like*) and *Ethylene response sensor 1* (*ERS1-like*), had similar expression levels in both control and NAA-treated fruits until the inception of ripening in the control (60 DAFB), when a significant increase was registered earlier in untreated berries than NAA-treated ones (Figure 5C and D). Three *Ethylene response factors* genes (*ERF3-like*, *ERF-AP2-like* and *ERF5-1*), involved in the regulation of ethylene response, were all positively affected by the NAA treatment, although with different timings (Figure 5E, F and G).

Since the HORMONOMETER analysis showed some significant variations also in the expression of abscisic acid (ABA) targets, two ABA-related genes were also investigated. The *9-cis-epoxycarotenoid dioxygenase3* (*NCED3*), which is a key gene involved in ABA biosynthesis, was significantly down-regulated in NAA-treated samples one week after the treatment (60 DAFB) (Figure 5H) and *Abscisic acid insensitive 3* (*ABI3*), involved in ABA perception, was down-regulated in NAA-treated berries up to 95 DAFB. Thereafter, its mRNA levels in NAA-treated samples reached those observed in untreated samples (Figure 5I).

## Discussion

A ripening delay caused by the application of auxins has been previously recorded both in climacteric and non-climacteric fruits [9,23]. In grapevine, a ripening delay induced by the application of natural or synthetic auxins, including NAA, to berries before véraison was observed in a large range of cultivars [6,11]. Results presented in this study confirmed that NAA, applied at the pre-véraison stage, strongly delays ripening inception in cv. Merlot (Figure 1). All the parameters used to monitor the ripening progression (in particular berry volume, SSC, and titratable acidity), with the exception of the initial delay occurring in the treated berries, showed overlapping kinetics in both treated and untreated fruit. These data suggest that the auxin treatment caused just a shift in the initiation of ripening, as already hypothesized by Böttcher et al. [7]. This observation is also confirmed by the microarray data analysis, which showed a decreasing number of differentially expressed genes throughout the experiment (see Additional file 2). At the véraison of control fruit (60 DAFB), MapMan analysis clearly shows that NAA application down-regulated genes involved in cell expansion (cell wall metabolism and water uptake) and secondary metabolism, in particular those responsible for flavonoids biosynthesis (Figure 2), consistently with the biochemical analyses. This repressive effect remained well evident up to 110 DAFB, whereas a partial recovery was observed thereafter, as already reported by Davies et al. [9] and Jeong et al. [11]. At harvest of NAA-treated berries (148 DAFB), the transcription level of genes involved in the flavonoids biosynthetic pathway was still enhanced with respect to the control fruit at harvest, while a full recovery was observed for those involved in cell wall metabolism and water uptake (see Additional file 7). These observations show that NAA is more effective in counteracting the accumulation of flavonoids rather than berry expansion, as demonstrated by Böttcher et al. [24].

Both in control and treated fruits there is a clear coordination of the transcriptional regulation of genes determining cell expansion (i.e. *EX1* and *PG1*) and turgor (i.e. *Pip1*), in agreement with the model for cell expansion proposed by Cosgrove [25], especially during the early post-treatment phases and up to 95 DAFB. During this phase, the NAA treatment clearly repressed the genes involved in both processes, which was consistent with the berry volume measures and thus reflecting an almost exclusive transcriptional control of berry expansion. An inversion of this trend and a complete recovery to the levels of the control was observed thereafter (after 95 DAFB), although not correlated with the faster volume increase occurring in treated berries to reach a final volume at harvest equal to that of the control fruits. This may be due to different mechanisms controlling berry expansion other than the auxin-controlled transcription, most likely at post-transcriptional level, as previously demonstrated for aquaporins whose gating behaviour can be affected by phosphorylation, heteromerization, pH, Ca^2+^, pressure, solute gradients and temperature [26]. Regulation of aquaporin trafficking may also represent a way to modulate membrane water permeability. Taken together, these data indicate that the berry expansion process is under the control of multiple regulatory pathways, involved according to a well-defined developmentally-programmed chronological sequence.

To shed light on the role of auxin and its cross-talk with other hormones in the regulation of berry ripening, a specific analysis was carried out on hormone-related genes by using the HORMONOMETER bioinformatic platform [27]. This was paralleled by a merged analysis of specific gene categories (i.e. the AHD categories). This approach allowed to set up a hypothetical model describing what happened in terms of auxin-related response after the NAA treatment (Figure 4). The application of NAA caused an excessive availability of auxin, most likely counterbalanced by homeostatic mechanisms involving synthesis, breakdown, conjugation and transport [28,29]. However, at 60 DAFB auxin biosynthesis and metabolism gene categories did not differ significantly between control and treated samples, transport was generally repressed, and an auxin-specific transcriptional response was seen along with a general activation of signal transduction elements. Therefore, it is likely that the homeostatic mechanisms had already been activated within the first 7 days after the treatment. This hypothesis is supported by qPCR expression data, especially those related to *GH3-like*, *IAA4-like*, and *IAA31-like* genes (Figure 5D, E, F). In NAA-treated berries at 57 DAFB, the first of these three auxin-related genes was expressed 6-fold higher compared to the control, then its expression decreased to just 2.5-fold at 60 DAFB, followed by a constantly decreasing trend leading to the same levels measured in the control at 95 DAFB. *GH3* (*Gretchen Hagen 3)* genes, specifically those belonging to group II [30], encode enzymes that conjugate IAA to amino acids. Interestingly, it has been recently shown that *GH3.1* plays a role in the formation of IAA-Aspartate at the onset of grape berry ripening, and it positively responds to the combined application of ABA and sucrose, and to ethylene, linking it to the control of ripening processes [24]. Nevertheless, both the *IAA* genes showed well-correlated diverging trends from 57 up to 60 DAFB, with the highest differences pointed out in the latter time point, coinciding also with the highest level of their expression in NAA-treated berries. Also the *ARF8-like* gene showed the largest divergence at 60 DAFB and the HORMONOMETER data indicate a very active transcriptional control compatible with an auxin-specific response. The expression patterns of these four genes along with the HORMONOMETER data and the overall physiological response indicate that biologically active concentrations of auxin were achieved throughout a homeostatic recovery occurring within 7 days after the treatment, during which the physiological response is mainly unspecific and due to a likely pharmacological effect of NAA. During this period, conjugation and transport may contribute to a decrease in the auxin levels, leading to the same range of concentration that can be found before ripening inception, thus generating a developmental block. This block is most likely mediated by a primary auxin signaling, whose main players include the *IAAs* and the *ARFs*, as their expression patterns indicate. At 110 DAFB, an overall repression of biosynthetic genes along with a stimulation of those coding for TR and ST elements was observed in NAA-treated samples. The HORMONOMETER indicates the activation of specific gene targets that were not auxin-specific, although they were compatible with still biologically active auxin levels. In this phase, a likely secondary homeostatic response was occurring, mainly at the level of biosynthesis as shown by the repression of upstream auxin BS genes such as *TRYPS-like*. The primary transcriptional response achieved within 110 DAFB triggered the recovery cascade that was active also thereafter, as demonstrated by biochemical parameters. However, at this stage the biological meaning of the homeostatic recovery is different from that occurring before 60 DAFB. It is likely that the early homeostatic reaction was just aimed at detoxifying from high auxin concentrations, whereas that occurring at 110 DAFB was a symptom of a normal ripening progression resembling the natural ripening inception during which auxin levels were shown to decrease [24]. Some auxin-specific targets, mainly TF-encoding, were shown to be active up to 148 DAFB, most likely triggering the transcriptional regulation of genes, such as *CHS1* and *F3H* that were shown to be down-regulated (see Additional file 7, A and C). At this stage, however, the overall transcriptional response was scarce since berry ripening was definitely accomplished, as shown by the physiological and biochemical parameters.

Fluctuations in auxin levels and response were shown to be correlated with ripening progression and a possible mechanism was hypothesized to explain how the berry reacts to the NAA treatment, but how does auxin action link to other hormones, such as ethylene, ABA, and brassinosteroids, that are known to regulate the same developmental processes?

The HORMONOMETER analysis may help to explain this aspect, especially considering the first comparison (N1/C1), in which the existence of a strong antagonistic effect between auxin and ethylene and, to a lesser extent, a substantial ‘synergism’ between auxin and ABA were shown. Both these aspects were quite marked for both the whole subset of transcriptional indexes (H) and the specific ones (sRG). The transient positive effect of NAA on the transcription of *ACS6* and *ACO2* genes (Figure 5A and B), already measured in other fruits [17,31,32], may be interpreted as a part of the secondary homeostatic reaction to the auxin treatment, as described above. As such, the transient increase of ethylene biosynthesis specifically induced by biologically active auxin concentrations would counteract the excess of auxin by activating downstream mechanisms, in this case related to the biosynthesis of the hormone (i.e. the *TRYPS* gene), thus releasing the berry from the developmental block.

According to the Arabidopsis model of ethylene signaling, reduced expression and activity of receptors increase sensitivity to ethylene, whereas increased receptor expression and activity decrease sensitivity [33]. It is also known that ethylene receptors act in cooperation, according to mutual, but often unique roles, thus differentially regulating ethylene responses and giving diverse outputs according to the receptor complex combination [34]. Furthermore, in Arabidopsis, EIN4 was shown to have a unique role in ethylene signaling [35,36] and a synergistic effect on ers1 function, as it is required to maintain ethylene insensitivity in an *ers1* background [34]. Taking into account these data, a relevant role during grape berry ripening may be played by the putative *AtEIN4* orthologue, as the corresponding gene was expressed in a ripening-dependent manner, with increasing levels after véraison, measured both in the control and NAA-treated samples (Figure 5C). Also an *ERS1*-like gene showed similar expression patterns, although shifted ahead (Figure 5D). Similar transcriptional behaviors were reported also by Deluc et al. [3] and Chervin and Deluc [37] along with a peak of ethylene biosynthesis, and may be consistent with a higher sensitivity to the hormone at véraison (delayed by the auxin treatment), which decreases thereafter throughout ripening.

The effect of auxin on genes involved in ethylene response was very weak, as seen in both the AHD and the HORMONOMETER analyses (Figure 3), with the exception of an *ERF5*-1 gene, which was significantly up-regulated at 60 DAFB (Figure 5G). A significant correlation was observed between the expression patterns of this gene and *ACS6*, leading to the hypothesis that *ERF5*-1 may mediate the auxin-induced up-regulation of ethylene biosynthetic genes in grape*.* This hypothesis is currently being investigated with dedicated experimental trials in order to shed light on the crosstalk between these two hormones, which is crucial for grape berry development and ripening.

Although the NAA treatment caused a general stimulation of ethylene biosynthesis and action, a negative effect on the transcription of genes involved in flavonoids biosynthesis, cell wall metabolism and water uptake, previously shown to be ethylene-related [12,14], was observed. Several studies have examined the interactions between auxin and ethylene at the transcriptional level and different models were proposed [38-40]. Taking into account this information, the effect of NAA may have bypassed the primary level of crosstalk between the two hormones, resulting into the activation of only some targets in common with ethylene that may belong to the secondary crosstalk. Consistent with this possibility, the upstream regulatory regions of many genes induced by auxin and ethylene were shown to contain putative auxin response element (AuxRE) and ethylene response element (ERE) sequences, which are sites for ARF and EIN3/EIL binding, respectively [39]. Future studies should specifically address this aspect.

The existence of a synergism between auxin and ABA was unexpected taking into account the opposite roles previously claimed for these hormones in the regulation of grape berry ripening [5]. These data, however, may indicate that the HORMOMETER analysis is able to reveal a previously unappreciated selectivity of auxin towards the regulation of ABA-related processes, as already reported by Volodarsky et al. [27] for salicylic acid and auxin. In fact, data presented here pointed out that auxin down-regulated the genes involved in ABA biosynthesis (Figure 5H), while the signal transduction pathway elements were substantially unaffected or stimulated (see Additional file 2). These ambiguous outcomes were already pointed out in previous studies revealing that ABA and auxin signaling pathways belong to a very complex regulatory network with unexpected features [41].

## Conclusions

**Figure 6:**
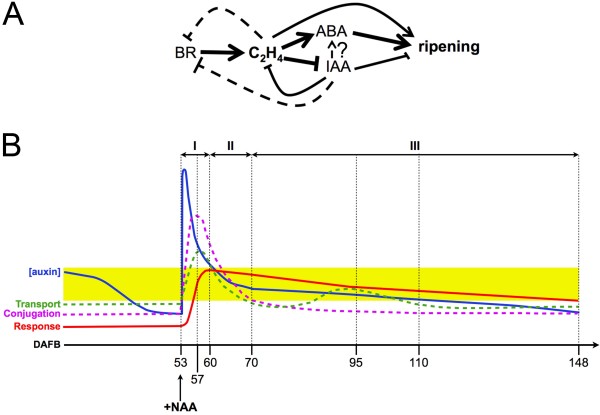
**Hypothetical model summarizing the interactions occurring between the hormones mainly involved in the regulation of ripening inception and progression. A**. Brassinosteroids and ethylene may trigger the first molecular events associated with ripening inception, with the latter hormone involved in the developmental shift preceding véraison. Ethylene would also negatively regulate auxin action by repressing its biosynthesis and trigger ABA-related genes in order to enable the progression of ripening-associated biochemical changes. A direct positive effect of ethylene on ripening may also be postulated based upon available data. Conventional symbols are used to describe positive and negative interactions. The thickness of the lines indicates the preferential ways of interactions determining ripening inception and progression, whereas dotted lines indicate possible feedback interactions. Interactions occurring between auxin and ABA are complex and still under investigation. **B**. Hypothetical model explaining auxin-related events occurring upon the NAA treatment (+NAA). This model was assembled based upon the expression of auxin-related genes. The yellow-shaded area indicates a likely range of auxin concentration compatible with its biological activity. Three main responsive phases were identified according to this model: phase I (53-60 DAFB), during which the berry displays a primary homeostatic response most likely due to an unspecific pharmacological reaction; phase II (60-70 DAFB), during which a biologically active concentration of auxin is recovered and a secondary homeostatic response is triggered, and phase III (70-148 DAFB), in which a normal ripening progression is observed.

Taking into account the available data concerning the hormonal regulation of the ripening syndrome in grape and tomato, a putative model was herein assembled to better understand the hormonal cross-talk occurring during our experiments (Figure 6). According to this working model, which is currently being validated, brassinosteroids (BR) may start the cascade of events leading to ripening by increasing ethylene levels, as reported in tomato [42]. It is known that a dramatic increase in endogenous BR levels occurs at the onset of fruit ripening in grape [8] and that also an ethylene peak is measurable just before véraison [43]. Moreover, ethylene seems to repress BR-regulated genes once ripening is triggered (Tonutti et al.*,* unpublished data), thus indicating a possible feedback mechanism allowing a time progression of the syndrome through the coordination of the downstream events. According to this view, ethylene may play a central role in ripening inception. On one hand, it acts independently and directly on the activation of ripening-associated processes, such as those related to cell wall modifications [12], and on the other hand it cooperates with ABA to indirectly trigger several biochemical changes associated with ripening, such as berry coloration [14,15]. It also represses auxin biosynthesis, thus releasing the berry from the developmental block exerted by this hormone [7]. When the NAA treatment was performed, the berry was most likely undergoing this developmental shift controlled by ethylene, which was still reversible. Therefore, the transient increase in auxin levels imposed by the exogenous treatment caused a reversion by counterbalancing the developmental control exerted by ethylene, thus leading the berry back to the pre-véraison stage with a consequent delay of the ripening progression.

## Methods

### Plant materials and treatment

Experiments were performed on *Vitis vinifera* L. cv. Merlot berries collected at a commercial vineyard (Vini e vigne, Monselice PD, Italy). One-hundred bunches from fifty homogeneous plants (two bunches per plant) were treated *in planta* with a synthetic auxin (naphtalenacetic acid, NAA, 200 mg/L; SIGMA-N640) at the pre-véraison stage corresponding to fifty-three days after full bloom (DAFB), as suggested by Jeong et al. [11]. Whole berries from treated and untreated bunches were collected at 57, 60, 70, 95, and 110 DAFB (see Additional file 10), and either immediately used for biochemical analyses or frozen in liquid nitrogen and stored at -80°C for RNA isolation and transcriptomic evaluations. Because of a delayed ripening observed upon the treatment, additional samples were collected from NAA-treated bunches up to 160 DAFB. The sample at 148 DAFB was chosen *ex post* as being representative of the harvest date of treated berries, according not only to the Color Index for Red Grape (CIRG), but also to the biochemical parameters that were similar to the control samples at harvest (see Results section for a detailed description). At each time-point, three biological replicates were sampled for the biochemical analyses and two for transcriptomic assessments. Each replicate was collected from five to seven bunches and was made up of at least fifty berries chosen according to the CIRG index proposed by Carreño et al. [44] at the same position within the cluster (median position). The juice from each replicate was used to assess the biochemical indicators (titratable acidity, pH, tartaric acid, malic acid, soluble solids) using a WineScan FT 120 multiple-parameter analyser (FOSS, Denmark), while anthocyanin content was determined as described by Mattivi et al. [45]. A colorimetric index was chosen since gene expression analyses in individual grape berries during ripening initiation revealed that pigmentation intensity could be assumed as a valid indicator of developmental staging within the cluster [46].

### RNA extraction, microarray analysis and quantitative real-time PCR

Total RNA for both microarray and real-time PCR experiments was extracted from whole berries stored at -80°C using the perchlorate method as reported by Rizzini et al. [47].

Microarray experiments were carried out using the grape AROS V1.0 platform (http://www.operon.com), as described by Rizzini et al. [47]. The following samples were hybridized: NAA-treated berries at 60 DAFB versus untreated berries at 60 DAFB (N1/C1), NAA-treated berries at 110 DAFB versus untreated berries at 110 DAFB (N2/C2), and NAA-treated berries at 148 DAFB versus untreated berries at 110 DAFB (N3/C2). For each of the three comparisons, three slides were hybridized using targets corresponding to two biological replicates (at least one biological replicate was dye-swapped, except for the N1/C1 comparison for which both replicates were dye-swapped and thus four slides were hybridized).

Raw hybridization data were quality-filtered, background-subtracted, and intra-array normalized with the *loess* method. The above calculations were all carried out with the package *limma* and other basic statistical functions of R for Mac OS X v2.13.1 (http://www.r-project.org/). The same package was also used for discovering differentially expressed genes by means of the linear modelling approach (*lmFit*) and the empirical Bayes statistics (*eBayes*), both implemented in *limma*[48].

All the experimental procedures comply with minimum information about a microarray experiment (MIAME) standards for array data [49]. Gene expression data have been submitted to Gene Expression Omnibus (GEO) (accession no. GSE37341) at NCBI (https://www.ncbi.nlm.nih.gov/geo/).

For quantitative real-time PCR analysis (qPCR), cDNA was synthesized using 2 μg of total RNA, 2.5 μM (dT)18 primer, 200 Units of M-MLV Reverse Transcriptase (Promega) and 1 Unit of RNAguard (Amersham Biosciences), at 37°C for 90 minutes in a final volume of 20 μL. qPCR was carried out in triplicate, on two biological replicates for each sample, with StepOne Plus Real-Time PCR System (Applied Biosystems) by using specific primers listed in Additional file 11. The specificity of amplification was assessed as indicated by Botton et al. [50]. Data were acquired, elaborated, and exported with the StepOne Software version 2.1 (Applied Biosystems), whereas all the final calculations were carried out with the automated Excel spreadsheet Q-Gene designed by Simon [51] using the modifications of the delta cycle threshold method suggested by Pfaffl [52]. Gene expression values were normalized to the housekeeping gene *UbiCF* (*Ubiquitin Conjugating Factor*; CF203457) already used by Castellarin et al. [53] and reported as arbitrary units of mean normalized expression, using equation 2 of Q-Gene.

### Microarray annotation and enrichment analysis

The sequences of the oligos spotted onto the AROS V1.0 microarray were matched by means of the Blastn algorithm against the transcripts of the 12X genome assembly obtained at the CRIBI Centre of the University of Padova and publicly available at the website http://genomes.cribi.unipd.it/. The Gene Ontology terms were retrieved, imported in the Blast2GO software v2.5.0 [54] and increased of about 16% by means of the Annex function [55] as reported by Botton et al. [20]. Enrichment analysis was performed for each set of differentially expressed genes with the built-in Fisher’s exact test function with *P* ≤ 0.01 and FDR correction.

### HORMONOMETER analyses

The HORMONOMETER tool (http://genome.weizmann.ac.il/hormonometer/) [27] was used by following the same pipeline adopted in peach by Bonghi et al. [22]. Since this bioinformatic tool accepts only Arabidopsis gene expression data, the probes spotted onto the grape microarray were matched against the 12X genome assembly as reported above and, in turn, the genes predicted in the latter release were matched with those of Arabidopsis by blasting the respective protein sequences against each other (grape deduced proteins vs TAIR10 proteins). In this way, an association ‘array probe-grape gene-Arabidopsis gene’ was obtained, allowing to use as input data for HORMONOMETER the grape gene expression data coupled with the respective locus names and Affymetrix probe IDs of the putative Arabidopsis orthologs. In the case in which different grape genes matched a single Arabidopsis gene, their expression values were averaged and considered just once. In addition to the whole set of grape genes spotted onto the microarray, three subsets were submitted to HORMONOMETER: i) genes with hormone-specific responsiveness (i.e. that are not multiple targets of hormones), ii) hormone-responsive genes encoding transcription factors (TFs), and iii) genes encoding TFs with hormone-specific responsiveness (an intersection between the two previous groups). A short description of the basic principles of functioning of the HORMONOMETER tool is given by Bonghi et al. [22].

## Competing interests

The authors do not have any competing interest.

## Authors’ contributions

CB and FMR devised the study and participated in its design and coordination; FMR and AR collected fruit material and conducted the microarray experiments; FZ and MC performed the validation of microarray data by qRT-PCR; CB, AB and MC analyzed the data and interpreted the results; CB, AB, AR, MC and FZ wrote the paper. All authors read and approved the final manuscript.

## Supplementary Material

Additional file 1 **(Figure S1A_B.pdf).** A. Parameters considered for sample selection. Analytical and transcriptional parameters, assessed as indicated in Materials and methods, considered for the selection of samples analysed in the microarray experiment. **B**. Ripening progression of control and NAA-treated fruit. Changes in fruit development and pigmentation at first comparison (60 DAFB), second comparison (110 DAFB) and third comparison (148 DAFB). Click here for file

Additional file 2 **(Table S1.xlsx).** List of genes differentially expressed in N1/C1, N2/C2 and N3/C2 comparisons. Click here for file

Additional file 3 **(Figure S2.jgp).** Validation of microarray data by quantitative real-time PCR (qPCR). In order to validate microarray gene expression data, quantitative real-time PCR (qPCR) was performed on fifteen genes whose IDs on the microarray are: Vv_10000861, Vv_10003711, Vv_10010895, Vv_10004167, Vv_10010748, Vv_10010857, Vv_10001614, Vv_10002511, Vv_10011058, Vv_10007514, Vv_10010764, Vv_10005187, Vv_10001287, Vv_10005087, Vv_10004370. Correlation plot and Pearson correlation index were calculated between microarray (X axis) and qPCR (Y axis) log ratios, only for genes with differential expression on the microarray experiment supported by statistically significant *P* values (*P* ≤ 0.05). Genes with non-significant statistics (i.e. *P* > 0.05) were discarded from this analysis. Click here for file

Additional file 4 **(Table S2.pdf).** Enriched GO terms of genes differentially expressed in N1/C1 comparison. For each term, the GO identifier (GO-ID), the complete Gene Ontology term (Term), the GO category to which it belongs (C = cellular component; F = molecular function; P = biological process), the FDR-corrected P-value and the P-value of the Fisher’s exact test, the number of sequences in the test set and in the background set annotated (#Test and #Ref) and not annotated (#notAnnotTest and #notAnnotRef) with the related GO term, the results of the test (Over- or Under-represented) and the percentages in the two sets are also given. Green and red background colours indicate under- or over-representation, respectively. Click here for file

Additional file 5 **(Table S3.pdf).** Enriched GO terms of genes differentially expressed in N2/C2 comparison. For each term, the GO identifier (GO-ID), the complete Gene Ontology term (Term), the GO category to which it belongs (C = cellular component; F = molecular function; P = biological process), the FDR-corrected P-value and the P-value of the Fisher’s exact test, the number of sequences in the test set and in the background set annotated (#Test and #Ref) and not annotated (#notAnnotTest and #notAnnotRef) with the related GO term, the results of the test (Over- or Under-represented) and the percentages in the two sets are also given. Green and red background colours indicate under- or over-representation, respectively. Click here for file

Additional file 6 **(Table S4.pdf).** Enriched GO terms of genes differentially expressed in N3/C2 comparison. For each term, the GO identifier (GO-ID), the complete Gene Ontology term (Term), the GO category to which it belongs (C = cellular component; F = molecular function; P = biological process), the FDR-corrected *P*-value and the P-value of the Fisher’s exact test, the number of sequences in the test set and in the background set annotated (#Test and #Ref) and not annotated (#notAnnotTest and #notAnnotRef) with the related GO term, the results of the test (Over- or Under-represented) and the percentages in the two sets are also given. Green and red background colours indicate under- or over-representation, respectively. Click here for file

Additional file 7 **(Figure S3.jgp).** Expression pattern, evaluated by qPCR, of genes involved in water uptake, polyphenols and cell wall metabolism. Expression pattern, evaluated by qPCR, of genes involved in water uptake (*TIP1;2-like*, Vv_10003817 and *AQUA1*, Vv_10003711), polyphenols (*CHS1*, Vv_10010748; *CHS3*, Vv_10004167; *F3H*, Vv_10003855; *UFGT*, Vv_10004481, *MYB31*, Vv17s0000g06190 and *MYB4*, Vv4s0023g03710) and cell wall metabolism (*PG1*, Vv_10003791 and *EX1*, Vv_10000426). Transcript levels in NAA-treated (square) and control (circle) berries are shown as means of normalized expression ±SE. Click here for file

Additional file 8 **(Table S5.pdf).** Categorization of genes showing significant change in their expression by using the MapMan platform. Categorization of genes showing significant change in their expression by using the MapMan platform. BinCode, BinName and Description are reported for each gene. Click here for file

Additional file 9 **(table S6.pdf).** Number of hormone-related genes in Arabidopsis and grape. Number of hormone-related genes in Arabidopsis and grape. For the latter species, information is reported concerning both the whole genome (see the genome release in the Materials and Methods section) and the AROS v1.0 microarray. Click here for file

Additional file 10 **(Figure S4.jgp).** Schematic representation of the experimental trial. Schematic representation of experimental trial with respect to the berry growth kinetics. The most relevant developmental phases are also indicated. The auxin treatment (NAA) was performed at 53 DAFB, whereas the sampling dates of both treated and untreated berries were at 57 (T1), 60 (T2), 70 (T3), 95 (T4), 110 (T5), and 148 (T6) DAFB. Samples used for the microarray analysis are indicated with black background labels. Click here for file

Additional file 11 **(Table S7.pdf).** Complete list of the primers sequences used in quantitative real-time PCR experiments. Click here for file
