# Supplementary material for: Grape berry ripening delay induced by a pre-véraison NAA treatment is paralleled by a shift in the expression pattern of auxin- and ethylene-related genes
Source: BMC Plant Biol. 2012 Oct 9;12:185. doi: 10.1186/1471-2229-12-185 (PMC3564861; doi:10.1186/1471-2229-12-185)
Supplement: Additional file 1 — (Figure S1A_B.pdf). A. Parameters considered for sample selection. Analytical and transcriptional parameters, assessed as indicated in Materials and methods, considered for the selection of samples analysed in the microarray experiment. B. Ripening progression of control and NAA-treated fruit. Changes in fruit development and pigmentation at first comparison (60 DAFB), second comparison (110 DAFB) and third comparison (148 DAFB). [file 1471-2229-12-185-S1.pdf]

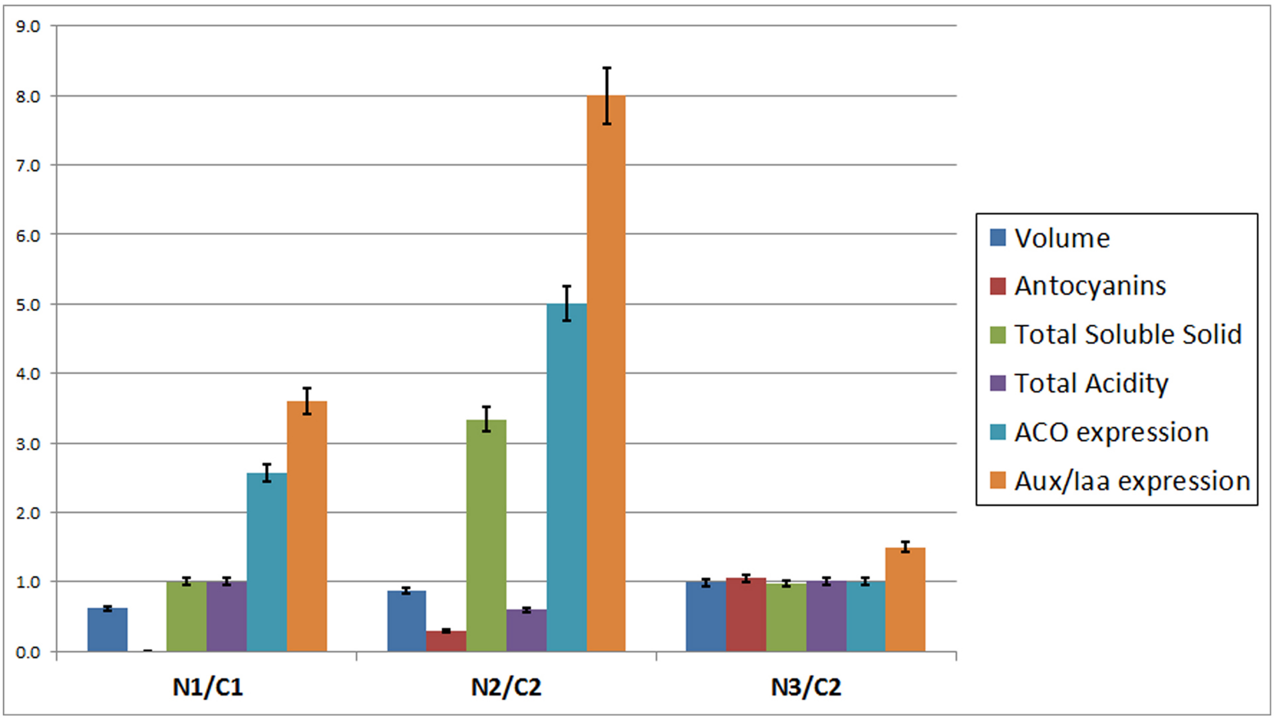

Figure S1A. Parameters considered for sample selection

Analytical and transcriptional parameters, assessed as indicated in Materials and methods, considered for the selection of samples analysed in the microarray experiment.

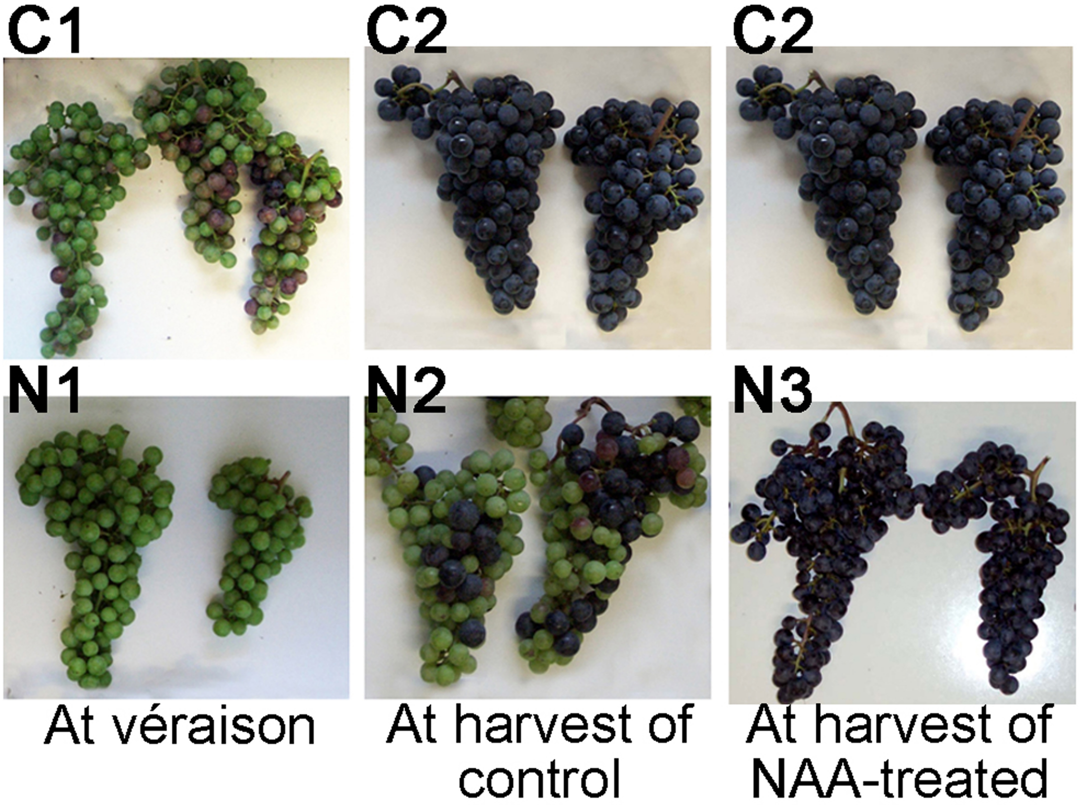

Figure S1B. Ripening progression of control and NAA-treated fruit

Changes in fruit development and pigmentation at first comparison (60 DAFB), second comparison (110 DAFB) and third comparison (148 DAFB).
