# Supplementary figures and images for: Grape berry ripening delay induced by a pre-véraison NAA treatment is paralleled by a shift in the expression pattern of auxin- and ethylene-related genes
Source: BMC Plant Biol. 2012 Oct 9;12:185. doi: 10.1186/1471-2229-12-185 (PMC3564861; doi:10.1186/1471-2229-12-185)

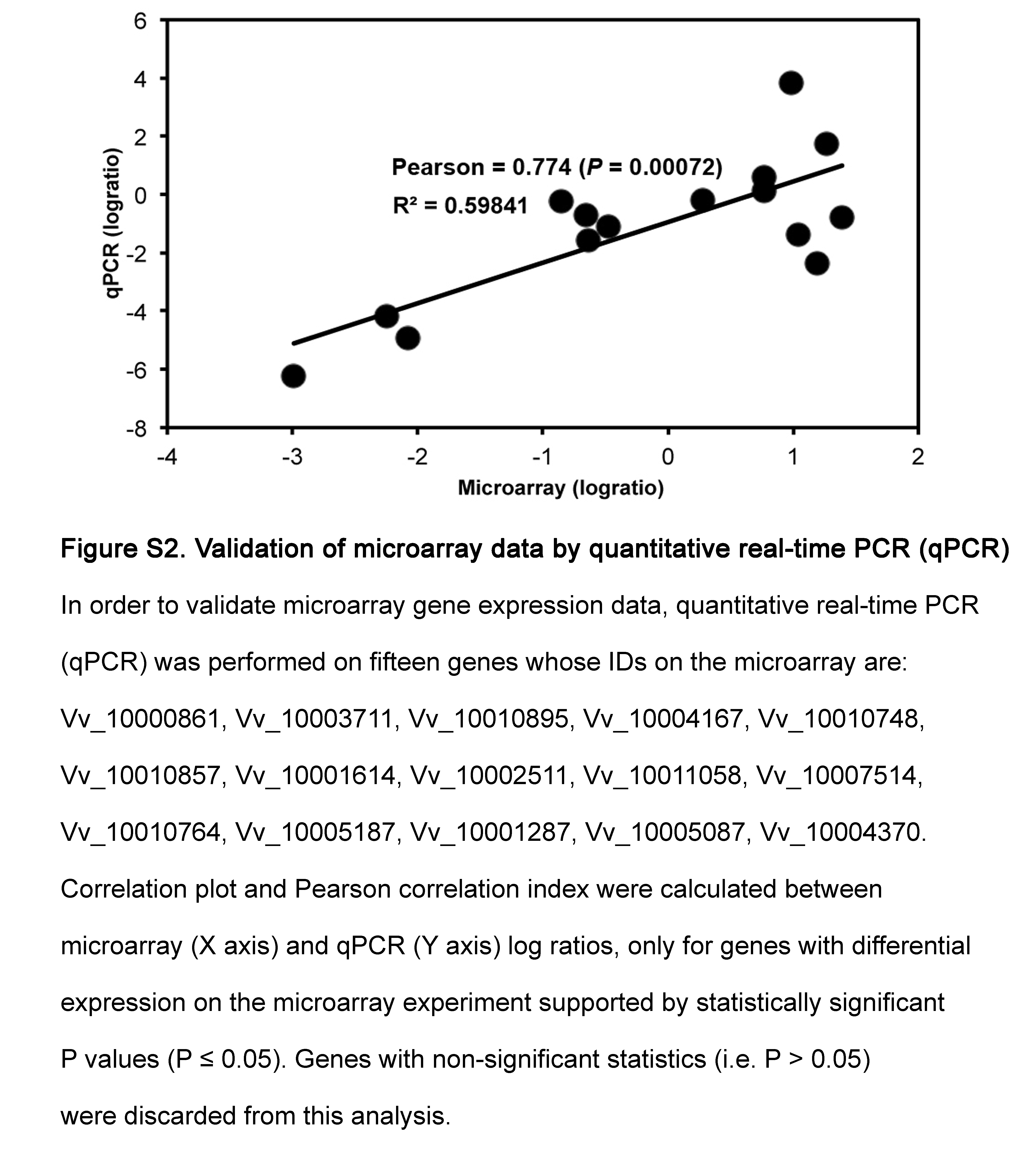

Supplement: Additional file 3 — (Figure S2.jgp). Validation of microarray data by quantitative real-time PCR (qPCR). In order to validate microarray gene expression data, quantitative real-time PCR (qPCR) was performed on fifteen genes whose IDs on the microarray are: Vv_10000861, Vv_10003711, Vv_10010895, Vv_10004167, Vv_10010748, Vv_10010857, Vv_10001614, Vv_10002511, Vv_10011058, Vv_10007514, Vv_10010764, Vv_10005187, Vv_10001287, Vv_10005087, Vv_10004370. Correlation plot and Pearson correlation index were calculated between microarray (X axis) and qPCR (Y axis) log ratios, only for genes with differential expression on the microarray experiment supported by statistically significant P values (P ≤ 0.05). Genes with non-significant statistics (i.e. P > 0.05) were discarded from this analysis. [file 1471-2229-12-185-S3.jpeg]

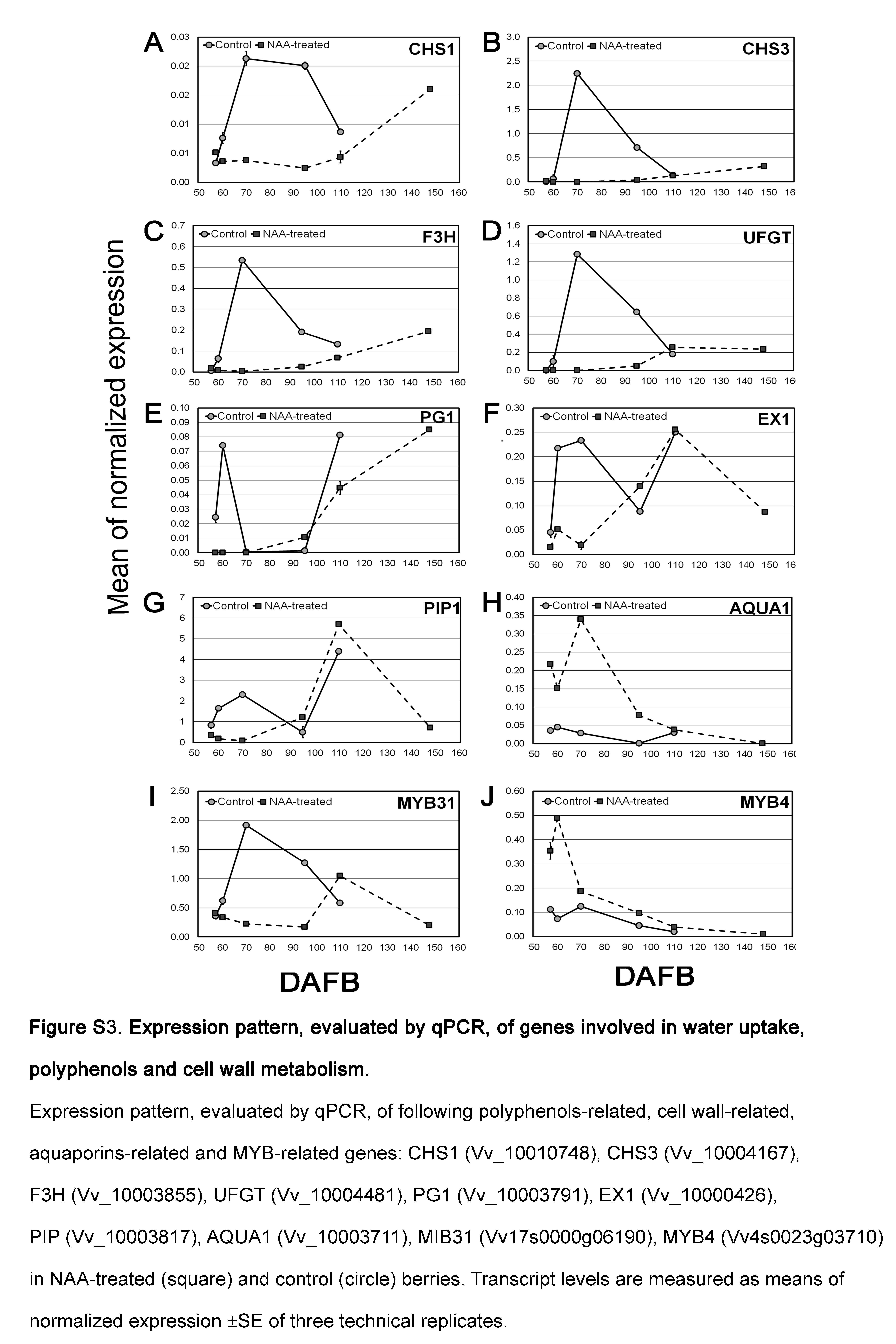

Supplement: Additional file 7 — (Figure S3.jgp). Expression pattern, evaluated by qPCR, of genes involved in water uptake, polyphenols and cell wall metabolism. Expression pattern, evaluated by qPCR, of genes involved in water uptake (TIP1;2-like, Vv_10003817 and AQUA1, Vv_10003711), polyphenols (CHS1, Vv_10010748; CHS3, Vv_10004167; F3H, Vv_10003855; UFGT, Vv_10004481, MYB31, Vv17s0000g06190 and MYB4, Vv4s0023g03710) and cell wall metabolism (PG1, Vv_10003791 and EX1, Vv_10000426). Transcript levels in NAA-treated (square) and control (circle) berries are shown as means of normalized expression ±SE. [file 1471-2229-12-185-S7.jpeg]

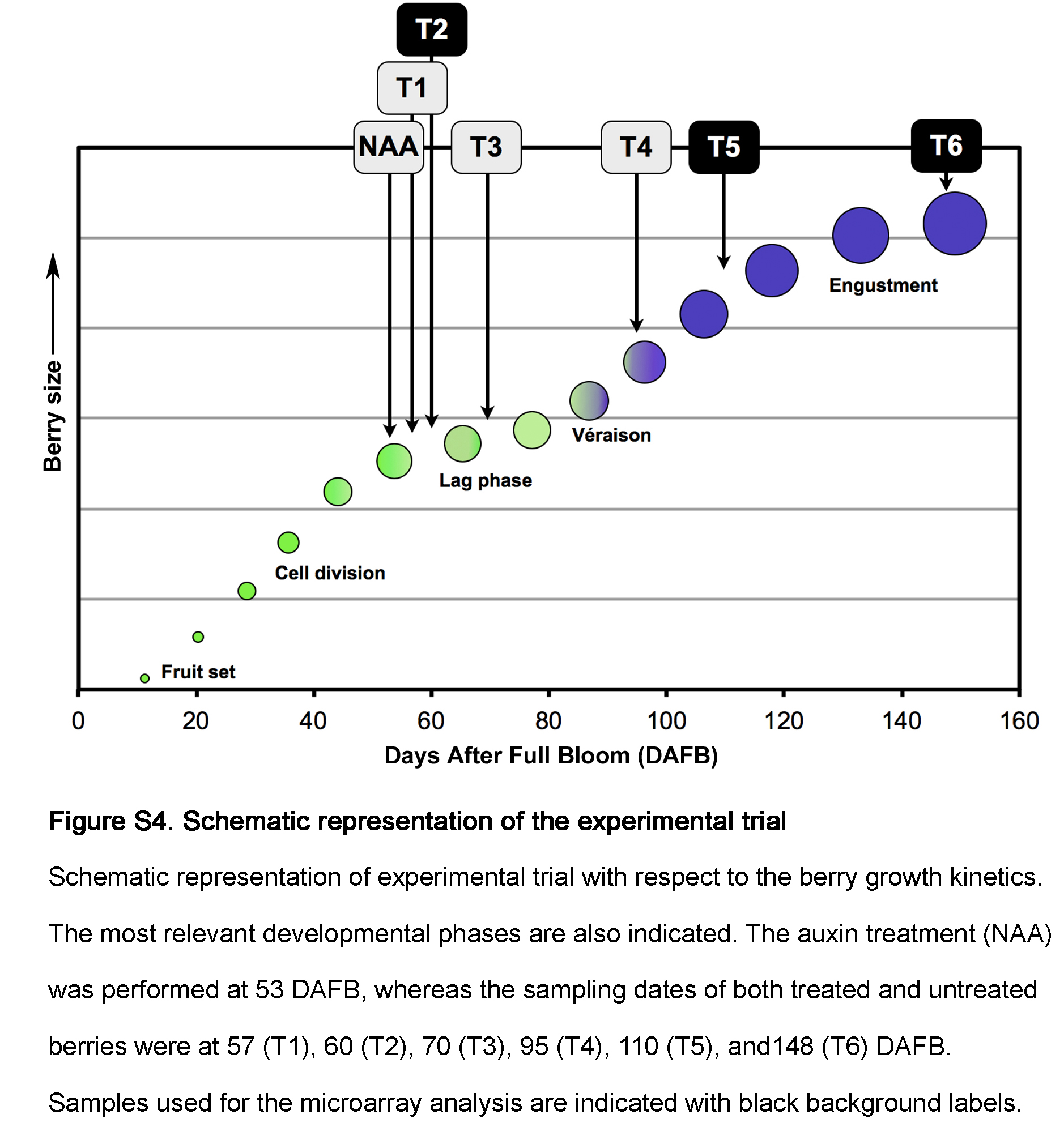

Supplement: Additional file 10 — (Figure S4.jgp). Schematic representation of the experimental trial. Schematic representation of experimental trial with respect to the berry growth kinetics. The most relevant developmental phases are also indicated. The auxin treatment (NAA) was performed at 53 DAFB, whereas the sampling dates of both treated and untreated berries were at 57 (T1), 60 (T2), 70 (T3), 95 (T4), 110 (T5), and 148 (T6) DAFB. Samples used for the microarray analysis are indicated with black background labels. [file 1471-2229-12-185-S10.jpeg]
