# Supplementary material for: Grape berry ripening delay induced by a pre-véraison NAA treatment is paralleled by a shift in the expression pattern of auxin- and ethylene-related genes
Source: BMC Plant Biol. 2012 Oct 9;12:185. doi: 10.1186/1471-2229-12-185 (PMC3564861; doi:10.1186/1471-2229-12-185)
Supplement: Additional file 4 — (Table S2.pdf). Enriched GO terms of genes differentially expressed in N1/C1 comparison. For each term, the GO identifier (GO-ID), the complete Gene Ontology term (Term), the GO category to which it belongs (C = cellular component; F = molecular function; P = biological process), the FDR-corrected P-value and the P-value of the Fisher’s exact test, the number of sequences in the test set and in the background set annotated (#Test and #Ref) and not annotated (#notAnnotTest and #notAnnotRef) with the related GO term, the results of the test (Over- or Under-represented) and the percentages in the two sets are also given. Green and red background colours indicate under- or over-representation, respectively. [file 1471-2229-12-185-S4.pdf]

Table S2. Enriched GO terms of genes differentially expressed in the comparison N1/C1. For each term, the GO identifier (GO-ID), the complete Gene Ontology term (Term), the GO category to which it belongs (C = cellular component; F = molecular function; P = biological process), the FDR-corrected *P*-value and the P-value of the Fisher's exact test, the number of sequences in the test set and in the background set annotated (#Test and #Ref) and not annotated (#notAnnotTest and #notAnnotRef) with the related GO term, the results of the test (Over- or Under-represented) and the percentages in the two sets are also given. Green and red background colours indicate under- or over-representation, respectively.

GO-ID	Term	Category	FDR-corrected P-value	P-Value	#Test	#Ref	#notAnnotTest	#notAnnotRef	Over/Under	% in test group	% in reference group
GO:0015031	protein transport	P	1	0,0012	17	216	1150	6768	under	1,46%	3,09%
GO:0045184	establishment of protein localization	P	1	0,0012	17	216	1150	6768	under	1,46%	3,09%
GO:0030529	ribonucleoprotein complex	C	1	0,0026	42	397	1125	6587	over	3,60%	5,68%
GO:0071843	cellular component biogenesis at cellular level	P	1	0,0026	26	279	1141	6705	over	2,23%	3,99%
GO:0070568	guanylyltransferase activity	F	1	0,0029	3	0	1164	6984	over	0,26%	0,00%
GO:0032991	macromolecular complex	C	1	0,0030	154	1160	1013	5824	over	13,20%	16,61%
GO:0044444	cytoplasmic part	C	1	0,0035	223	1602	944	5382	over	19,11%	22,94%
GO:0009628	response to abiotic stimulus	P	1	0,0035	43	152	1124	6832	over	3,68%	2,18%
GO:0005737	Cytoplasm	C	1	0,0039	286	1998	881	4986	over	24,51%	28,61%
GO:0009408	response to heat	P	1	0,0044	15	36	1152	6948	over	1,29%	0,52%
GO:0008104	protein localization	P	1	0,0051	20	223	1147	6761	over	1,71%	3,19%
GO:0004518	nuclease activity	F	1	0,0051	3	75	1164	6909	under	0,26%	1,07%
GO:0006412	Translation	P	1	0,0052	41	379	1126	6605	over	3,51%	5,43%
GO:0005840	Ribosome	C	1	0,0055	34	327	1133	6657	over	2,91%	4,68%
GO:0022613	ribonucleoprotein complex biogenesis	P	1	0,0056	25	258	1142	6726	over	2,14%	3,69%
GO:0006886	intracellular protein transport	P	1	0,0065	9	128	1158	6856	under	0,77%	1,83%
GO:0022803	passive transmembrane transporter activity	F	1	0,0068	0	35	1167	6949	under	0,00%	0,50%
GO:0015267	channel activity	F	1	0,0068	0	35	1167	6949	under	0,00%	0,50%
GO:0022838	substrate-specific channel activity	F	1	0,0068	0	35	1167	6949	under	0,00%	0,50%
GO:0005216	ion channel activity	F	1	0,0068	0	35	1167	6949	under	0,00%	0,50%
GO:0042254	ribosome biogenesis	P	1	0,0070	25	256	1142	6728	over	2,14%	3,67%
GO:0005198	structural molecule activity	F	1	0,0070	25	256	1142	6728	over	2,14%	3,67%
GO:0051649	establishment of localization in cell	P	1	0,0074	14	170	1153	6814	under	1,20%	2,43%
GO:0003735	structural constituent of ribosome	F	1	0,0080	20	215	1147	6769	over	1,71%	3,08%
GO:0046907	intracellular transport	P	1	0,0089	12	150	1155	6834	under	1,03%	2,15%
GO:0008235	metalloexopeptidase activity	F	1	0,0091	6	8	1161	6976	over	0,51%	0,11%
