# Supplementary material for: Grape berry ripening delay induced by a pre-véraison NAA treatment is paralleled by a shift in the expression pattern of auxin- and ethylene-related genes
Source: BMC Plant Biol. 2012 Oct 9;12:185. doi: 10.1186/1471-2229-12-185 (PMC3564861; doi:10.1186/1471-2229-12-185)
Supplement: Additional file 5 — (Table S3.pdf). Enriched GO terms of genes differentially expressed in N2/C2 comparison. For each term, the GO identifier (GO-ID), the complete Gene Ontology term (Term), the GO category to which it belongs (C = cellular component; F = molecular function; P = biological process), the FDR-corrected P-value and the P-value of the Fisher’s exact test, the number of sequences in the test set and in the background set annotated (#Test and #Ref) and not annotated (#notAnnotTest and #notAnnotRef) with the related GO term, the results of the test (Over- or Under-represented) and the percentages in the two sets are also given. Green and red background colours indicate under- or over-representation, respectively. [file 1471-2229-12-185-S5.pdf]

Table S3. Enriched GO terms of genes differentially expressed in the comparison N2/C2. For each term, the GO identifier (GO-ID), the complete Gene Ontology term (Term), the GO category to which it belongs (C = cellular component; F = molecular function; P = biological process), the FDR-corrected *P*-value and the P-value of the Fisher's exact test, the number of sequences in the test set and in the background set annotated (#Test and #Ref) and not annotated (#notAnnotTest and #notAnnotRef) with the related GO term, the results of the test (Over- or Under-represented) and the percentages in the two sets are also given. Green and red background colours indicate under- or over-representation, respectively.

GO-ID	Term	Category	FDR-corrected P-value	P-Value	#Test	#Ref	#notAnnotTest	#notAnnotRef	Over/Under	% in test group	% in reference group
GO:0006412	Translation	P	0,003	6,33E-07	14	406	769	6962	under	1,79%	5,51%
GO:0030529	ribonucleoprotein complex	C	0,03	1,28E-05	18	421	765	6947	under	2,30%	5,71%
GO:0044267	cellular protein metabolic process	P	0,05	4,48E-05	92	1279	691	6089	over	11,75%	17,36%
GO:0005840	Ribosome	C	0,05	4,89E-05	14	347	769	7021	under	1,79%	4,71%
GO:0071843	cellular component biogenesis at cellular level	P	0,08	9,38E-05	11	294	772	7074	under	1,40%	3,99%
GO:0022613	ribonucleoprotein complex biogenesis	P	0,08	1,25E-04	10	273	773	7095	under	1,28%	3,71%
GO:0042254	ribosome biogenesis	P	0,10	1,75E-04	10	271	773	7097	under	1,28%	3,68%
GO:0019538	protein metabolic process	P	0,10	1,89E-04	116	1495	667	5873	over	14,81%	20,29%
GO:0034660	ncRNA metabolic process	P	0,16	4,01E-04	1	105	782	7263	under	0,13%	1,43%
GO:0009059	macromolecule biosynthetic process	P	0,16	4,23E-04	68	955	715	6413	over	8,68%	12,96%
GO:0003735	structural constituent of ribosome	F	0,16	4,27E-04	8	227	775	7141	under	1,02%	3,08%
GO:0042592	homeostatic process	P	0,20	5,92E-04	1	101	782	7267	under	0,13%	1,37%
GO:0051258	protein polymerization	P	0,20	6,57E-04	8	14	775	7354	over	1,02%	0,19%
GO:0006399	tRNA metabolic process	P	0,20	6,82E-04	0	76	783	7292	under	0,00%	1,03%
GO:0006072	glycerol-3-phosphate metabolic process	P	0,24	8,83E-04	3	0	780	7368	over	0,38%	0,00%
GO:0004564	beta-fructofuranosidase activity	F	0,28	0,001082134	4	2	779	7366	over	0,51%	0,03%
GO:0005737	Cytoplasm	C	0,30	0,001257003	181	2103	602	5265	over	23,12%	28,54%
GO:0005739	Mitochondrion	C	0,35	0,001523937	13	275	770	7093	under	1,66%	3,73%
GO:0043228	non-membrane-bounded organelle	C	0,36	0,001739096	38	581	745	6787	over	4,85%	7,89%
GO:0043232	intracellular non-membrane-bounded organelle	C	0,36	0,001739096	38	581	745	6787	over	4,85%	7,89%
GO:0010467	gene expression	P	0,44	0,002253978	62	845	721	6523	over	7,92%	11,47%
GO:0007047	cellular cell wall organization	P	0,44	0,002412551	24	108	759	7260	over	3,07%	1,47%
GO:0045229	external encapsulating structure organization	P	0,44	0,002521314	24	109	759	7259	over	3,07%	1,48%
GO:0071555	cell wall organization	P	0,44	0,002804715	24	111	759	7257	over	3,07%	1,51%
GO:0030312	external encapsulating structure	C	0,44	0,002804715	24	111	759	7257	over	3,07%	1,51%
GO:0019725	cellular homeostasis	P	0,44	0,00281955	1	86	782	7282	under	0,13%	1,17%
GO:0070882	cellular cell wall organization or biogenesis	P	0,52	0,003405478	26	127	757	7241	over	3,32%	1,72%
GO:0003677	DNA binding	F	0,52	0,003533901	92	629	691	6739	over	11,75%	8,54%
GO:0034645	cellular macromolecule biosynthetic process	P	0,54	0,003811078	62	832	721	6536	over	7,92%	11,29%
GO:0046246	terpene biosynthetic process	P	0,58	0,004312989	4	4	779	7364	over	0,51%	0,05%
GO:0009522	photosystem I	C	0,58	0,004556977	7	16	776	7352	over	0,89%	0,22%
GO:0005198	structural molecule activity	F	0,58	0,005241502	14	267	769	7101	under	1,79%	3,62%
GO:0019866	organelle inner membrane	C	0,58	0,005435754	2	98	781	7270	under	0,26%	1,33%
GO:0045454	cell redox homeostasis	P	0,58	0,006020713	1	76	782	7292	under	0,13%	1,03%
GO:0016021	integral to membrane	C	0,58	0,006689388	124	911	659	6457	over	15,84%	12,36%
GO:0019899	enzyme binding	F	0,58	0,007177704	4	5	779	7363	over	0,51%	0,07%
GO:0009538	photosystem I reaction center	C	0,58	0,007613825	3	2	780	7366	over	0,38%	0,03%
GO:0044444	cytoplasmic part	C	0,58	0,007792551	146	1679	637	5689	over	18,65%	22,79%
GO:0005215	transporter activity	F	0,58	0,007834964	59	383	724	6985	over	7,54%	5,20%
GO:0044085	cellular component biogenesis	P	0,58	0,008234973	23	368	760	7000	over	2,94%	4,99%
GO:0009058	biosynthetic process	P	0,58	0,008344845	135	1567	648	5801	over	17,24%	21,27%
GO:0008299	isoprenoid biosynthetic process	P	0,58	0,008464348	13	51	770	7317	over	1,66%	0,69%

GO:0005618	cell wall	C	0,58	0,008627662	21	103	762	7265	over	2,68%	1,40%
GO:0043038	amino acid activation	P	0,58	0,008947951	0	55	783	7313	under	0,00%	0,75%
GO:0043039	tRNA aminoacylation	P	0,58	0,008947951	0	55	783	7313	under	0,00%	0,75%
GO:0006418	tRNA aminoacylation for protein translation	P	0,58	0,008947951	0	55	783	7313	under	0,00%	0,75%
GO:0016875	ligase activity, forming carbon-oxygen bonds	F	0,58	0,008947951	0	55	783	7313	under	0,00%	0,75%
GO:0016876	ligase activity, forming aminoacyl-tRNA and related compounds	F	0,58	0,008947951	0	55	783	7313	under	0,00%	0,75%
GO:0004812	aminoacyl-tRNA ligase activity	F	0,58	0,008947951	0	55	783	7313	under	0,00%	0,75%
GO:0009099	valine biosynthetic process	P	0,58	0,009206419	6	14	777	7354	over	0,77%	0,19%
GO:0009098	leucine biosynthetic process	P	0,58	0,009206419	6	14	777	7354	over	0,77%	0,19%
GO:0030418	nicotianamine biosynthetic process	P	0,58	0,009217221	2	0	781	7368	over	0,26%	0,00%
GO:0072350	tricarboxylic acid metabolic process	P	0,58	0,009217221	2	0	781	7368	over	0,26%	0,00%
GO:0072351	tricarboxylic acid biosynthetic process	P	0,58	0,009217221	2	0	781	7368	over	0,26%	0,00%
GO:0030417	nicotianamine metabolic process	P	0,58	0,009217221	2	0	781	7368	over	0,26%	0,00%
GO:0030410	nicotianamine synthase activity	F	0,58	0,009217221	2	0	781	7368	over	0,26%	0,00%
GO:0008615	pyridoxine biosynthetic process	P	0,58	0,009217221	2	0	781	7368	over	0,26%	0,00%
GO:0042819	vitamin B6 biosynthetic process	P	0,58	0,009217221	2	0	781	7368	over	0,26%	0,00%
GO:0008614	pyridoxine metabolic process	P	0,58	0,009217221	2	0	781	7368	over	0,26%	0,00%
GO:0009331	glycerol-3-phosphate dehydrogenase complex	C	0,58	0,009217221	2	0	781	7368	over	0,26%	0,00%
GO:0050306	sucrose 1F-fructosyltransferase activity	F	0,58	0,009217221	2	0	781	7368	over	0,26%	0,00%
GO:0047207	1,2-beta-fructan 1F-fructosyltransferase activity	F	0,58	0,009217221	2	0	781	7368	over	0,26%	0,00%
GO:0050738	fructosyltransferase activity	F	0,58	0,009217221	2	0	781	7368	over	0,26%	0,00%
GO:0048467	gynoecium development	P	0,58	0,009217221	2	0	781	7368	over	0,26%	0,00%
GO:0048440	carpel development	P	0,58	0,009217221	2	0	781	7368	over	0,26%	0,00%
