# Supplementary material for: Grape berry ripening delay induced by a pre-véraison NAA treatment is paralleled by a shift in the expression pattern of auxin- and ethylene-related genes
Source: BMC Plant Biol. 2012 Oct 9;12:185. doi: 10.1186/1471-2229-12-185 (PMC3564861; doi:10.1186/1471-2229-12-185)
Supplement: Additional file 6 — (Table S4.pdf). Enriched GO terms of genes differentially expressed in N3/C2 comparison. For each term, the GO identifier (GO-ID), the complete Gene Ontology term (Term), the GO category to which it belongs (C = cellular component; F = molecular function; P = biological process), the FDR-corrected P-value and the P-value of the Fisher’s exact test, the number of sequences in the test set and in the background set annotated (#Test and #Ref) and not annotated (#notAnnotTest and #notAnnotRef) with the related GO term, the results of the test (Over- or Under-represented) and the percentages in the two sets are also given. Green and red background colours indicate under- or over-representation, respectively. [file 1471-2229-12-185-S6.pdf]

**Table S4.** Enriched GO terms of genes differentially expressed in the comparison N3/C2. For each term, the GO identifier (GO-ID), the complete Gene Ontology term (Term), the GO category to which it belongs (C = cellular component; F = molecular function; P = biological process), the FDR-corrected *P*-value and the P-value of the Fisher's exact test, the number of sequences in the test set and in the background set annotated (#Test and #Ref) and not annotated (#notAnnotTest and #notAnnotRef) with the related GO term, the results of the test (Over- or Under-represented) and the percentages in the two sets are also given. Green and red background colours indicate under- or over-representation, respectively.

| GO-ID | Term | Category | FDR-corrected P-value | P-Value | #Test | #Ref | #notAnnotTest | #notAnnotRef | Over/Under | % in test group | % in reference group |
|---|---|---|---|---|---|---|---|---|---|---|---|
| GO:0032502 | developmental process | P | 0,02 | 4,29E-06 | 47 | 175 | 828 | 7101 | over | 5,37% | 2,41% |
| GO:0048856 | anatomical structure development | P | 0,05 | 3,84E-05 | 33 | 115 | 842 | 7161 | over | 3,77% | 1,58% |
| GO:0030529 | ribonucleoprotein complex | C | 0,05 | 4,71E-05 | 23 | 416 | 852 | 6860 | under | 2,63% | 5,72% |
| GO:0007275 | multicellular organismal development | P | 0,05 | 5,09E-05 | 40 | 155 | 835 | 7121 | over | 4,57% | 2,13% |
| GO:0044085 | cellular component biogenesis | P | 0,16 | 2,09E-04 | 21 | 370 | 854 | 6906 | under | 2,40% | 5,09% |
| GO:0005840 | Ribosome | C | 0,16 | 2,45E-04 | 19 | 342 | 856 | 6934 | under | 2,17% | 4,70% |
| GO:0022613 | ribonucleoprotein complex biogenesis | P | 0,16 | 2,69E-04 | 13 | 270 | 862 | 7006 | under | 1,49% | 3,71% |
| GO:0042254 | ribosome biogenesis | P | 0,19 | 3,66E-04 | 13 | 268 | 862 | 7008 | under | 1,49% | 3,68% |
| GO:0015079 | potassium ion transmembrane transporter activity | F | 0,20 | 4,32E-04 | 6 | 5 | 869 | 7271 | over | 0,69% | 0,07% |
| GO:0032501 | multicellular organismal process | P | 0,30 | 7,42E-04 | 44 | 203 | 831 | 7073 | over | 5,03% | 2,79% |
| GO:0009653 | anatomical structure morphogenesis | P | 0,34 | 0,001096599 | 15 | 44 | 860 | 7232 | over | 1,71% | 0,60% |
| GO:0003735 | structural constituent of ribosome | F | 0,34 | 0,001235975 | 11 | 224 | 864 | 7052 | under | 1,26% | 3,08% |
| GO:0006412 | Translation | P | 0,34 | 0,001511524 | 26 | 394 | 849 | 6882 | over | 2,97% | 5,42% |
| GO:0008360 | regulation of cell shape | P | 0,34 | 0,001656198 | 4 | 2 | 871 | 7274 | over | 0,46% | 0,03% |
| GO:0006075 | 1,3-beta-glucan biosynthetic process | P | 0,34 | 0,001656198 | 4 | 2 | 871 | 7274 | over | 0,46% | 0,03% |
| GO:0051273 | beta-glucan metabolic process | P | 0,34 | 0,001656198 | 4 | 2 | 871 | 7274 | over | 0,46% | 0,03% |
| GO:0051274 | beta-glucan biosynthetic process | P | 0,34 | 0,001656198 | 4 | 2 | 871 | 7274 | over | 0,46% | 0,03% |
| GO:0006074 | 1,3-beta-glucan metabolic process | P | 0,34 | 0,001656198 | 4 | 2 | 871 | 7274 | over | 0,46% | 0,03% |
| GO:0003843 | 1,3-beta-glucan synthase activity | F | 0,34 | 0,001656198 | 4 | 2 | 871 | 7274 | over | 0,46% | 0,03% |
| GO:0000148 | 1,3-beta-glucan synthase complex | C | 0,34 | 0,001656198 | 4 | 2 | 871 | 7274 | over | 0,46% | 0,03% |
| GO:0003006 | reproductive developmental process | P | 0,38 | 0,001961337 | 19 | 65 | 856 | 7211 | over | 2,17% | 0,89% |
| GO:0031047 | gene silencing by RNA | P | 0,55 | 0,00320588 | 6 | 9 | 869 | 7267 | over | 0,69% | 0,12% |
| GO:0030117 | membrane coat | C | 0,55 | 0,003483822 | 8 | 17 | 867 | 7259 | over | 0,91% | 0,23% |
| GO:0048475 | coated membrane | C | 0,55 | 0,003483822 | 8 | 17 | 867 | 7259 | over | 0,91% | 0,23% |
| GO:0022604 | regulation of cell morphogenesis | P | 0,55 | 0,003536476 | 4 | 3 | 871 | 7273 | over | 0,46% | 0,04% |
| GO:0016458 | gene silencing | P | 0,55 | 0,003537098 | 7 | 13 | 868 | 7263 | over | 0,80% | 0,18% |
| GO:0009908 | flower development | P | 0,55 | 0,004128227 | 9 | 22 | 866 | 7254 | over | 1,03% | 0,30% |
| GO:0005198 | structural molecule activity | F | 0,55 | 0,004227517 | 16 | 265 | 859 | 7011 | under | 1,83% | 3,64% |
| GO:0071843 | cellular component biogenesis at cellular level | P | 0,55 | 0,004450481 | 18 | 287 | 857 | 6989 | over | 2,06% | 3,94% |
| GO:0009867 | jasmonic acid mediated signaling pathway | P | 0,55 | 0,004537155 | 3 | 1 | 872 | 7275 | over | 0,34% | 0,01% |
| GO:0071395 | cellular response to jasmonic acid stimulus | P | 0,55 | 0,004537155 | 3 | 1 | 872 | 7275 | over | 0,34% | 0,01% |
| GO:0048608 | reproductive structure development | P | 0,55 | 0,004649039 | 17 | 61 | 858 | 7215 | over | 1,94% | 0,84% |
| GO:0006486 | protein amino acid glycosylation | P | 0,55 | 0,004669425 | 6 | 10 | 869 | 7266 | over | 0,69% | 0,14% |
| GO:0043413 | macromolecule glycosylation | P | 0,55 | 0,004669425 | 6 | 10 | 869 | 7266 | over | 0,69% | 0,14% |
| GO:0070085 | Glycosylation | P | 0,55 | 0,004821862 | 7 | 14 | 868 | 7262 | over | 0,80% | 0,19% |
| GO:0010629 | negative regulation of gene expression | P | 0,55 | 0,004821862 | 7 | 14 | 868 | 7262 | over | 0,80% | 0,19% |
| GO:0003725 | double-stranded RNA binding | F | 0,64 | 0,005852941 | 5 | 7 | 870 | 7269 | over | 0,57% | 0,10% |
| GO:0009791 | post-embryonic development | P | 0,64 | 0,005901754 | 17 | 64 | 858 | 7212 | over | 1,94% | 0,88% |
| GO:0006566 | threonine metabolic process | P | 0,71 | 0,006747297 | 10 | 29 | 865 | 7247 | over | 1,14% | 0,40% |
| GO:0044238 | primary metabolic process | P | 0,73 | 0,007095868 | 322 | 3024 | 553 | 4252 | over | 36,80% | 41,56% |
| GO:0010605 | negative regulation of macromolecule metabolic process | P | 0,81 | 0,008401734 | 7 | 16 | 868 | 7260 | over | 0,80% | 0,22% |
