# Supplementary material for: Grape berry ripening delay induced by a pre-véraison NAA treatment is paralleled by a shift in the expression pattern of auxin- and ethylene-related genes
Source: BMC Plant Biol. 2012 Oct 9;12:185. doi: 10.1186/1471-2229-12-185 (PMC3564861; doi:10.1186/1471-2229-12-185)
Supplement: Additional file 8 — (Table S5.pdf). Categorization of genes showing significant change in their expression by using the MapMan platform. Categorization of genes showing significant change in their expression by using the MapMan platform. BinCode, BinName and Description are reported for each gene. [file 1471-2229-12-185-S8.pdf]

Table S5. – Categorization of genes showing significant change in their expression by using the MapMan platform. BinCode, BinName and Description are reported for each gene.

Identifier	BinCode	BinName	Description
vv_10009170	1.1.1.1	PS.lightreaction.photosystem II.LHC-II	Light harvesting chlorophyll a/b-binding protein precursor
vv_10003952	1.1.1.1	PS.lightreaction.photosystem II.LHC-II	Chlorophyll a-b binding protein CP24 10A
vv_10006831	1.1.1.2	PS.lightreaction.photosystem II.PSII polypeptide subunits	Photosystem II protein W-like
vv_10005736	1.1.1.2	PS.lightreaction.photosystem II.PSII polypeptide subunits	photosystem II 10 kDa protein
vv_10008857	1.1.1.2	PS.lightreaction.photosystem II.PSII polypeptide subunits	Photosystem II reaction center W protein
vv_10001089	1.1.1.2	PS.lightreaction.photosystem II.PSII polypeptide subunits	Oxygen evolving enhancer protein 1 precursor
vv_10004084	1.1.2.2	PS.lightreaction.photosystem I.PSI polypeptide subunits	Photosystem I reaction center subunit III
vv_10000096	1.1.4	PS.lightreaction.ATP synthase	ATP synthase B' chain
vv_10010705	1.1.4	PS.lightreaction.ATP synthase	ATP synthase delta chain
vv_10006259	1.1.4	PS.lightreaction.ATP synthase	ATP synthase CF0 subunit III
vv_10009663	1.1.40	PS.lightreaction.cyclic electron flow-chlororespiration	NADH dehydrogenase I subunit N
vv_10010940	1.1.40	PS.lightreaction.cyclic electron flow-chlororespiration	NADH-plastoquinone oxidoreductase chain 1
vv_10011190	1.1.5.3	PS.lightreaction.other electron carrier (ox/red).ferredoxin reductase	Ferredoxin--NADP reductase leaf-type isozyme chloroplast precursor
CB004194	10.06.03	cell wall.degradation.pectate lyases and polygalacturonases	polygalacturonase-like protein
vv_10003897	10.7	cell wall.modification	xyloglucan endo-transglycosylase-like protein
vv_10004343	10.7	cell wall.modification	Expansin-like protein
vv_10003332	10.7	cell wall.modification	xyloglucan endotransglucosylase/hydrolase protein 16 precursor
vv_10009273	10.8.1	cell wall.pectin*esterases.PME	pectinesterase
vv_10002517	10.8.1	cell wall.pectin*esterases.PME	Pectinesterase-like protein
vv_10001257	10.8.1	cell wall.pectin*esterases.PME	Pectin methylesterase isoform alpha
vv_10008442	10.8.1	cell wall.pectin*esterases.PME	pectin methylesterase
vv_10003598	16.1.4	secondary metabolism.isoprenoids.carotenoids	Beta-carotene hydroxylase
vv_10002547	16.1.4	secondary metabolism.isoprenoids.carotenoids	Beta-carotene hydroxylase-like
vv_10000978	16.2.1.1	secondary metabolism.phenylpropanoids.lignin biosynthesis.PAL	phenylalanine ammonium lyase
vv_10008484	16.2.1.1	secondary metabolism.phenylpropanoids.lignin biosynthesis.PAL	phenylalanine ammonium lyase [Vitis vinifera]
vv_10002562	16.2.1.10	secondary metabolism.phenylpropanoids.lignin biosynthesis.CAD	Cinnamyl alcohol dehydrogenase
vv_10001454	16.4.1	secondary metabolism.N misc.alkaloid-like	Short chain alcohol dehydrogenase-like
vv_10010748	16.8.2	secondary metabolism.flavonoids.chalcones	Chalcone synthase
vv_10004167	16.8.2	secondary metabolism.flavonoids.chalcones	Chalcone synthase-like
vv_10001887	16.8.3	secondary metabolism.flavonoids.dihydroflavonols	cinnamoyl-CoA reductase
vv_10011797	16.8.3	secondary metabolism.flavonoids.dihydroflavonols	cinnamoyl-CoA reductase
