# Supplementary material for: Grape berry ripening delay induced by a pre-véraison NAA treatment is paralleled by a shift in the expression pattern of auxin- and ethylene-related genes
Source: BMC Plant Biol. 2012 Oct 9;12:185. doi: 10.1186/1471-2229-12-185 (PMC3564861; doi:10.1186/1471-2229-12-185)
Supplement: Additional file 9 — (table S6.pdf). Number of hormone-related genes in Arabidopsis and grape. Number of hormone-related genes in Arabidopsis and grape. For the latter species, information is reported concerning both the whole genome (see the genome release in the Materials and Methods section) and the AROS v1.0 microarray. [file 1471-2229-12-185-S9.pdf]

Table S6 – Number of hormone-related genes in Arabidopsis and grape. For the latter species, information is reported concerning both the whole genome (see the genome release in the Materials and Methods section) and the AROS v1.0 microarray.

	No. Arabidopsis hormone indexes (% of the whole transcriptome)[a]	No. grape hormone indexes (% of the whole t transcriptome)[b]	No. grape hormone indexes (% of the whole array)[c]
Auxin	2,260 (8.2%)	2,831 (9.4%)	1,078 (7.4%)
Cytokinin	2,493 (9.1%)	3,032 (10.1%)	1,274 (8.7%)
Gibberellin	2,329 (8.5%)	2,718 (9.1%)	1,085 (7.5%)
Abscisic acid	2,215 (8.1%)	2,707 (9.0%)	1,146 (7.9%)
Ethylene	2,566 (9.4%)	2,904 (9.7%)	1,173 (8.1%)
Jasmonate	1,900 (6.9%)	2,368 (7.9%)	943 (6.5%)
Salicylate	1,036 (3.8%)	1,637 (5.5%)	625 (4.3%)
Brassinosteroids	2,567 (9.4%)	2,941 (9.8%)	1,299 (8.9%)

a) Percentages calculated on a total of 27,416 protein coding genes;

b) Percentages calculated on a total of 29,972 protein coding genes;

c) Percentages calculated on a total of 14,562 array probes.
