# Supplementary material for: Grape berry ripening delay induced by a pre-véraison NAA treatment is paralleled by a shift in the expression pattern of auxin- and ethylene-related genes
Source: BMC Plant Biol. 2012 Oct 9;12:185. doi: 10.1186/1471-2229-12-185 (PMC3564861; doi:10.1186/1471-2229-12-185)
Supplement: Additional file 11 — (Table S7.pdf). Complete list of the primers sequences used in quantitative real-time PCR experiments. [file 1471-2229-12-185-S11.pdf]

Table S7 – Complete list of the primers sequences used in Real-Time PCR experiments.

Gene Target	Primer Name	Operon ID	*Vitis vinifera 12x*	Sequence (F: forward, R: reverse)
auxin response factor 8	ARF8	Vv_10003009	VIT_04s0079g00160	F: GCGATCGGTTAAGGTTGGTTGGGATGAG<br>R: CCAGGATGCCAGGGTCGTTTTAGTCTG
putative indole-3-acetic acid-amido synthetase GH3.9	GH3-like	Vv_10007966	VIT_07s0005g00090	F: GCTAGAAGAGTGTTGCATTGCTGTGGAAG<br>R: TCTCGAGCGGTCCTACAGACTTATCGTGT
AUX/IAA transcriptional regulator family protein 4	IAA4-like	Vv_10002615	VIT_14s0030g02310	F: ACTCTTTCGGCTTGGGTTCT<br>R: GGATCTTGGTGCCACAACTT
Indole-3-Acetic acid inducible 31	IAA31-like	Vv_10000794	VIT_05s0020g01070	F: GGAGCTCCGTATTTAAGAAAAGTTGATCTG<br>R: GCATCCAATCTCCATCCCTGTCCTCATAG
Arabidopsis PIN formed 3	PIN3-like	Vv_10007217	VIT_01s0011g04860	F: GAGTCCTCCTGCGCGTTGCCATTGTCCAG<br>R: CCCAAATATGACTCCTGTGCTGAGAATATC
Auxin-Fbox 5	AUX-FBOX5	Vv_10008404	VIT_18s0001g07120	F: CCCGTGGCTCGAGAAGGTTTACCTGAAG<br>R: CACTGGTACCGAAGCCGTCACAACAAA
tryptophan synthase beta-subunit 1	TRYPS-like	Vv_10007514	VIT_19s0177g00310	F: TCTGATGCATGCCCTCACCGAGCTGGA<br>R: CTCCCTGCCAACATAGTCCTTCAATATTCC
ethylene-forming enzyme	ACO	Vv_10004370	VIT_12s0059g01380	F: GACTCCGAGCCCACACTGATGCCG<br>R: GGAGGCCGCTGACCGTGTCGTCTTG
1-aminocyclopropane-1-carboxylic acid (acc) synthase 6	ACS	Vv_10001614	VIT_02s0025g00360	F: GAGGTGGAGAACAGGAGTGC<br>R: GAGCAAGCCTTTCACTTTGG
Signal transduction histidine kinase, hybrid-type, ethylene sensor	EIN4-like	Vv_10010357	VIT_14s0081g00630	F: GGAGGGTCTGAGCTTCAGCATGTGCAA<br>R: CTCGAAGTCCTCTGAACATAGAATTGGAA
ethylene responsive element binding factor 3	ERF3-like	Vv_10001775	VIT_12s0059g01460	F: GGTGTACCGAGCGGATCAGTTTCAGGAG<br>R: CTAAAGGACTCCACGGTGCTGCTCATG
Ethylene response factor 5	ERF 5-1	Vv_10001287	VIT_16s0013g00970	F: GGTGCAGATGGGAATGGTAT<br>R: AGACTGTAGCGCCGTCAGAT
Integrase-type DNA-binding superfamily protein	ERF-AP2 like	Vv_10000332	VIT_16s0013g00900	F: ATCTTCGGCCTCTGATTCAA<br>R: GAAGACGGCAGATGGAGAAG
ethylene response sensor 1	ERS1	Vv_10007917	VIT_07s0005g00850	F: CCGGACAAGGGCTGCACAGTTACATTCG<br>R: GATTTGTTCGACCCCTTGGTACAGCTGC
Transport inhibitor response 1	TIR1-like	Vv_10005087	VIT_07s0104g01320	F: GACTCCGAGTTGAGGCTGTCTTGATGAAT<br>R: CCCTTGTTACCCATTTCTACCCCAACC
ABI3-interacting protein 2	ABI3	Vv_10001065	VIT_04s0008g02290	F: GAGATCCTGGACAATGATACTAGAGCCCC<br>R: TCACCACTGAGCTTTGATAAGGCGTCTTC
Nine-cis-epoxycarotenoid dioxygenase 3	NCED3-like	Vv_10009127	VIT_19s0093g00550	F: TCAGAGCTGCAGATAGTGAACGCCACGA<br>R: GTGGAATCCATAGGGAACTCGTGAGGGA
Chalcone synthase 1	CHS1	Vv_10010748	VIT_14s0068g00930	F: GAAGCCAGTGAAGCAGGTAGCCAACAA<br>R: CTCAGCTCTCTGGGCCTTTCTAATTTCTG
Chalcone synthase 3	CHS3	Vv_10004167	VIT_05s0136g00260	F: GAAGTCGGCTGAGGAAGGGCTGAAGACC<br>R: TCAACAGTGAGCCCTGGTCCGAAAC
Flavanone 3-hydroxylase	F3H	Vv_10003855	VIT_04s0023g03370	F: CGATCCGGGAACCATCACGCTGC<br>R: GTGATCCAAGTCTTTCCGCCATCC
UDP-glucose:flavonoid 3-O-glucosyltransferase	UFGT	Vv_10004481	VIT_16s0039g02230	F: GCAGTTTGGGGCTTTTGTAACACATTGT<br>R: CCAAAAAAGGGCCTGCAAATCAAG
Putative aquaporin TIP3	AQUA1	Vv_10003711	VIT_06s0061g00730	F: GGGACATGGCCTTGGACTGTTTGTGG<br>R: CACCGACGAAGGCTCCAAATGTGACAG
Tonoplast intrinsic protein	PIP	Vv_10003817	VIT_08s0007g04780	F: GGGAATTATTGCACCGATTG<br>R: AGGCCCAAATGATACTGCTG
Cellulase synthase	CS	Vv_10010895	VIT_00s1349g00010	F: TGGTCATCAACACAGTTCTCTCAGTCATGG<br>R: GAGAGCATAAAATGTTAGACATGACCCACC
Expansin1	EX1	Vv_10000426	VIT_01s0026g02620	F: GTCTTTTACCGCCCGCCACTAGTATCTGA<br>R: TGAAGGCATAACATCTGAGGGCACATTC
Polygalatturonase1	PG1	Vv_10003791	VIT_08s0007g08330	F: GGTGCACCAACGGCCTTAACCTTCTAT<br>R: CGAAGCTTGAACACCCACGCATTTTTC
Xyloglucan endotransglucosylases/hydrolases	XTH	Vv_10010764	VIT_11s0052g01230	F: GACAGACTGGTCGCAAGCTCCCTTCA<br>R: AAACTGAGCTGCAAGAGGATTTCCCAG
MYB domain protein 4 *	MYB4	/	VIT_04s0023g03710	F: CCTCTCCGATTCTGCAAGTG<br>R: GAAGAAGCAGGAATGGCAATG
MYB domain Protein 31 *	MYB31	/	VIT_17s0000g06190	F: GGGCAACTACACCAGATGCT<br>R: AGAGAATGGTTTCGGGTGTG

**Provided by Alessandro Vannozzi, DAFNAE Department*
